# Long-term durability of immune responses to the BNT162b2 and mRNA-1273 vaccines based on dosage, age and sex

**DOI:** 10.1038/s41598-022-25134-0

**Published:** 2022-12-08

**Authors:** Chapin S. Korosec, Suzan Farhang-Sardroodi, David W. Dick, Sameneh Gholami, Mohammad Sajjad Ghaemi, Iain R. Moyles, Morgan Craig, Hsu Kiang Ooi, Jane M. Heffernan

**Affiliations:** 1 Modelling Infection and Immunity Lab, Mathematics and Statistics, York University, 4700 Keele St, Toronto, ON M3J 1P3 Canada; 2 Centre for Disease Modelling, Mathematics and Statistics, York University, 4700 Keele St, Toronto, ON M3J 1P3 Canada; 3 Department of Mathematics, University of Manitoba, 186 Dysart Road, Winnipeg, MB R3T 2N2 Canada; 4 Digital Technologies Research Centre, National Research Council Canada, 222 College Street, Toronto, ON M5T 3J1 Canada; 5 Department of Mathematics and Statistics, Université de Montréal & Sainte-Justine University Hospital Research Centre, 3175, ch. Côte Sainte-Catherine, Montréal, QC H3T 1C5 Canada

**Keywords:** Systems biology, Diseases, Mathematics and computing, Immunology, Vaccines

## Abstract

The lipid nanoparticle (LNP)-formulated mRNA vaccines BNT162b2 and mRNA-1273 are a widely adopted multi vaccination public health strategy to manage the COVID-19 pandemic. Clinical trial data has described the immunogenicity of the vaccine, albeit within a limited study time frame. Here, we use a within-host mathematical model for LNP-formulated mRNA vaccines, informed by available clinical trial data from 2020 to September 2021, to project a longer term understanding of immunity as a function of vaccine type, dosage amount, age, and sex. We estimate that two standard doses of either mRNA-1273 or BNT162b2, with dosage times separated by the company-mandated intervals, results in individuals losing more than 99% humoral immunity relative to peak immunity by 8 months following the second dose. We predict that within an 8 month period following dose two (corresponding to the original CDC time-frame for administration of a third dose), there exists a period of time longer than 1 month where an individual has lost more than 99% humoral immunity relative to peak immunity, regardless of which vaccine was administered. We further find that age has a strong influence in maintaining humoral immunity; by 8 months following dose two we predict that individuals aged 18–55 have a four-fold humoral advantage compared to aged 56–70 and 70+ individuals. We find that sex has little effect on the immune response and long-term IgG counts. Finally, we find that humoral immunity generated from two low doses of mRNA-1273 decays at a substantially slower rate relative to peak immunity gained compared to two standard doses of either mRNA-1273 or BNT162b2. Our predictions highlight the importance of the recommended third booster dose in order to maintain elevated levels of antibodies.

## Introduction

The severe acute respiratory syndrome coronavirus 2 (SARS-CoV-2) first detected in December of 2019 has driven COVID-19 into a global pandemic^1–3^. Recognizing the severity of this novel pneumonia outbreak, global research efforts were rapidly organized and the first viral sequence became publicly available on 26 December 2019^4^. Immediately, various vaccine research groups began optimizing their current vaccine technologies to express the wildtype SARS-CoV-2 spike protein. SARS-CoV-2 vaccine candidates then resulted, including inactivated virus, viral protein subunits, messenger RNA (mRNA) recombinant human adenovirus, and non-viral replicating vector vaccines. Among these vaccine candidates, mRNA vaccines had a head start in exploiting the viral sequence due to its inherent rapid prototyping (high yield in-vitro transcription reactions) and manufacturing scalability^5^. The pre-clinical trial was initiated immediately in January 2020 and in April 2020, Phase I/II of the clinical trials commenced. In less than 8 months, the mRNA vaccines BNT162b2 (manufactured by Pfizer-BioNTech) and mRNA-1273 (manufactured by Moderna) were approved for emergency use in several countries^6,7^.

As of July 22, 2022, 86.16% (31.3 million individuals) of the 5-year and older population in Canada has received a primary series of vaccinations, with Pfizer-BioNTech and Moderna comprising 50.54% (19.3 million people) and 14.13% (5.4 million people) of vaccinated individuals, respectively^8^. Further, more than 357 and 227 million doses of Pfizer-BioNTech and Moderna vaccines, respectively, have been administered in the United States^9^. On July 29th, 2021, the Ministry of Health of Israel announced a third-booster-dose strategy based on preliminary report finding vaccine efficacy in prevention of infection via the Pfizer-BioNTech vaccine drops from 75 to 16% seven months following the second dose^10^. There is therefore urgency to understand and accurately predict waning immunity amongst individuals who received two-doses of the LNP-formulated mRNA vaccines, and to provide an estimate for a correlate of protection.

For acute SARS-CoV-2 infection, antibody and memory B-cell responses have been reported to be robust for up to 8 months^11^, and T-cell responses have been shown to be robust up 12 months following infection by the wild type strain^12^. In comparison, two doses of mRNA-1273 and BNT162b2 have shown seroconversion in the short term that surpasses that of recovered patients: 1 month post second dose, antibody stimulation (IgG and IgM) is reported to be higher while neutralizing antibodies are shown to be similar to recovered patients for fully vaccinated individuals^13–15^. In vaccinated SARS-CoV-2 naive individuals, studies of antibodies responses are predominantly short-(less than 60 days) and medium-term (less than 120 days)^11,16–23^, with one recent study examining antibody response out to day 210^24^. With the continued emergence of new variants of concern, there is a key gap in the understanding of the long-term (beyond 120 days) robustness of the two-dose LNP-formulated mRNA vaccines adopted by health authorities. To address this gap, we established an ODE-based mechanistic model that describes the humoral immune response of mRNA vaccines to predict long term immunity. We fit our model to reported antibody and cytokine levels to clinical trial data using two standard doses of mRNA-1273 (100 $\upmu$g) or BNT162b2 (30 $\upmu$g)^18–23^, as well as separately to two low doses of mRNA-1273 (25 $\upmu$g)^25^. Our mechanistic modelling approach allows us to gain insight into the biological processes involved in the mRNA vaccine uptake process in humans. Model results show significant decay in antibody a few months after dose-two inoculation, with the decay rate depending on the vaccine type, dose size, and age of the vaccine recipient. We find little difference between male and female predicted vaccine response.

## Results

**Figure 1:**
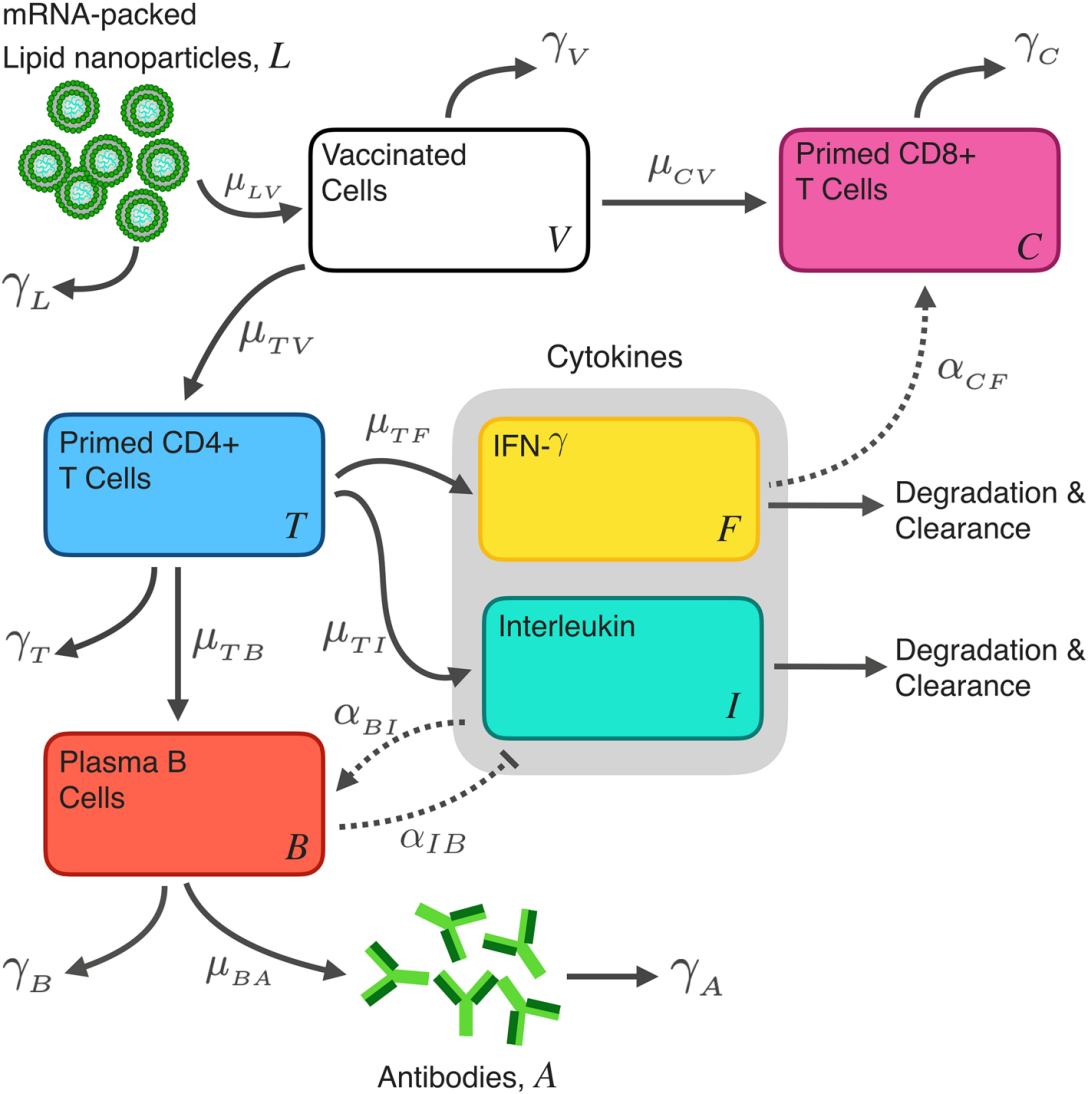
Schematic of the within-host model for inocculation and subsequent immunity generated from LNP-formulated mRNA vaccines. The full model description and mathematical assumptions can be found in “Model for in-host mRNA vaccination” section.

The details of all standard two-dose BNT162b2 and mRNA-1273 IgG and cytokine data sets used in this study can be found in in Table 3. All two-dose data sets were simultaneously fit to our model (Eq. 9) (shown schematically in Fig. 1) using the non-linear mixed-effects algorithm ‘SAEM’ (Stochastic Approximation Expectation-Maximization) in Monolix^26^ (see “Parameter estimation, fitting assessment, and long-term simulations” section for further details). All model fits are biologically informed; fixed parameters are determined by literature-informed sources, and parameter values found by the fitting routine lie within acceptable ranges found in the literature (see Table 1).

The population fit parameters for the two-standard-dose results are shown in Table S2, with all individual fit parameters shown in Tables S3 and S4. Plots of individual fits to each data set are shown in Figures S1–S4. Predictive checks and parameter distributions are included in Figs. S5–S6 and distributions of the random effects in Figures S7–S8. In this work we also consider a comparison to two low doses (25 $\upmu$g) of mRNA-1273. The population parameter fit values to the low dose data can be found in Table S5, while all individual fit values can be found in Tables S6–S7. Plots for individual fits to the two low doses of mRNA-1273 data can be found in Fig. S9, goodness of fit and predictive checks are shown in Fig. S10, and distributions of random effects are provided in Figs. S11–12. A Partial Rank Correlation Coefficient sensitivity analysis is performed to assess how model fit parameters affect the peak response from each state variable, the result of which is shown in Figure S13. We find the sensitivity analysis to reveal intuitive trends based on the model structure; peak state variable responses are sensitive to their respective source and removal terms.

**Table 1:** Samples of literature-sourced half-lives to compare with our SARS-CoV-2 two-dose mRNA-vaccine values.

Parameter	Definition	Fitted value ($\hbox {d}^{-1}$)	Equivalent half life	Half life value sourced from the literature
$\gamma _{A}$	IgG degradation rate	0.042 (mRNA-1273), 0.043 (BNT162b2)	$\sim$16 days	36 days (IgG) $\dagger$^29^, 21 days (IgG) $\dagger$^30^, 20.4 days (NAb) $\dagger$^31^, 68.8 days (anti-S titres)^32^
$\gamma _{V}$	Antigen presenting cell decay rate	0.07	10 days	10 days (mice)$*$ $\dagger$^33^
$\gamma _{F}$	IFN-$\gamma$ clearance rate	201.24	5 min	30 min (human)^34^, less than 3 min (mice)^35^, 40 min (mice)^36^
$\gamma _{I}$	Interleukin clearance	0.027	25.7 days	15.5 h (IL-6, mice)^37^, 2.5-5 h (IL-15, human)^38^, 5 h (IL-8, human)^39^
$\gamma _{B}$	Plasma B cell degradation rate	0.071	9.8 days	11.6 days (human)$\dagger$^40^, 7-8 weeks (mice)^41^, 18 days (human)^42^

Literature sourced values are not necessarily SARS-CoV-2-specific, rather are listed here to demonstrate that the model-determined values are correct to within an order of magnitude of other values found in the literature. $*$ This value is not a half life, but is the total observed time of translation. $\dagger$ These values are SARS-CoV-2 specific.

### Model is consistent with clinically-observed humoral responses

**Figure 2:**
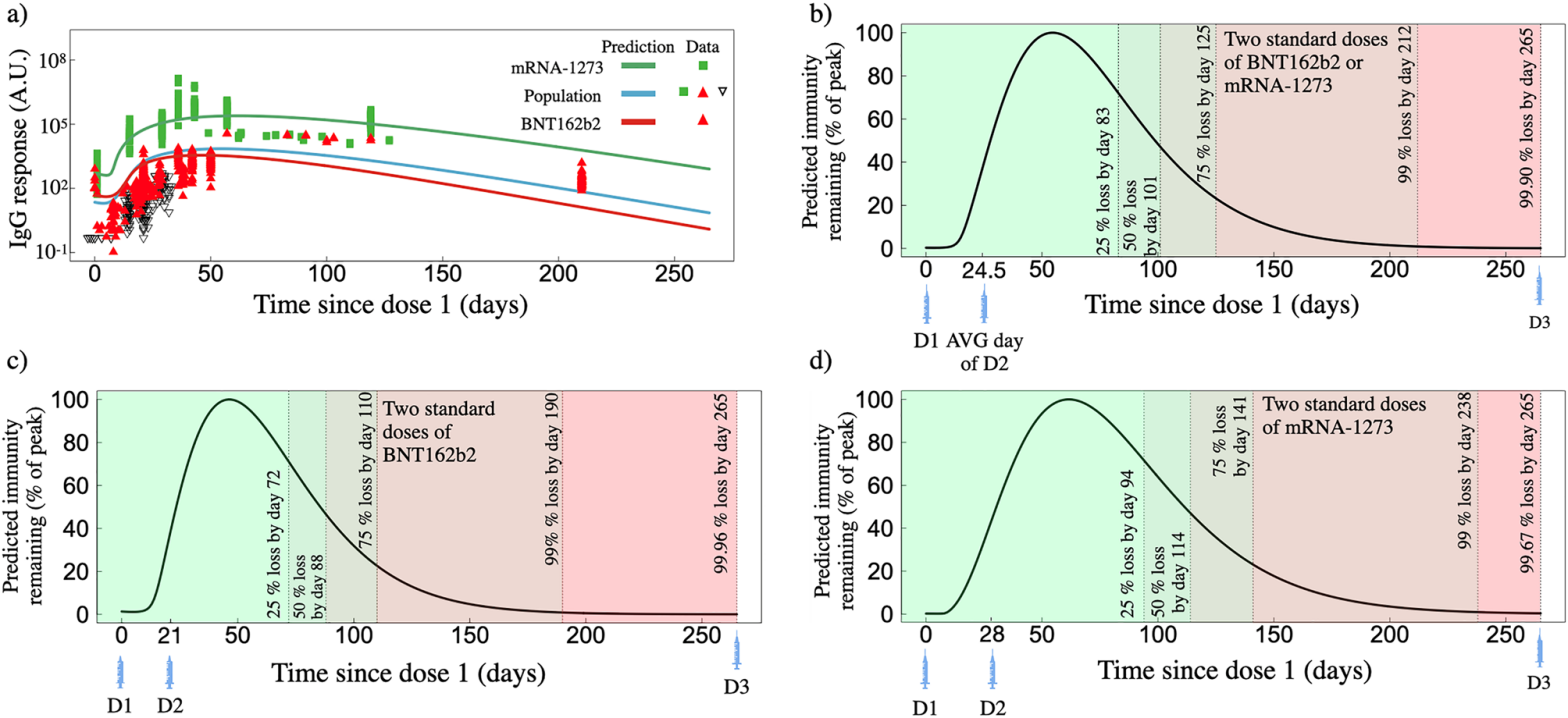
Predicted IgG response based on fits to clinical data for two standard doses of BNT162b2 or mRNA-1273. (**a**) IgG as a function of time since dose one for two standard doses of mRNA-1273 (green line, green squares represent actual data), BNT162b2 (red line, red triangles represent actual data), and the population fit which uses both mRNA-1273 and BNT162b2 two-dose data (blue line, combines all available data). (**b**) Predicted IgG remaining, normalized by the peak IgG count, for the population fit (panel **a**, blue line) as a function of days since the first dose. (**c**) Predicted IgG remaining for the two-dose BNT162b2 fit (corresponding to red line in Fig. 1a) as a function of days since the first dose. (**d**) Predicted IgG remaining for the two-dose mRNA-1273 fit (corresponding to green line in Fig. 1a) as a function of days since the first dose.

In Fig. 2 we summarize our findings for the IgG responses from two standard doses of BNT162b2 or mRNA-1273. The average standard two-dose model IgG response for mRNA-1273 (determined by the individual fits to the clinical data obtained from^22,23^, see Tables S3 and S4) and BNT162b2 (determined by the fits to the clinical data obtained from^19–21,24^, see Tables S3 and S4) vaccines are shown in Fig. 2a and are denoted by green and red dots, respectively (average are determined using the appropriate studies corresponding to each vaccine—see Tables S3 and S4). The population fit taking into account all standard two-dose data from BNT162b2 and mRNA-1273 is shown in blue. We show our long-term predicted response for each two-standard-dose vaccine type up to day 265, which marks the initial approximate date of the planned dose three, 8 months following dose two^27^, which, however, was later adjusted to a 6 month post-dose-two timeline^28^.

We show our long-term predicted response for each two-standard-dose vaccine type up to day 265, which marks the initial approximate date of the planned dose three, 8 months following dose two^27^, however, was later adjusted to a 6 month post-dose-two timeline^28^.

We note that for all IgG responses, the y-axes are in arbitrary units (A.U.) and depend on the methodology employed within each respective publication. In this work each clinical data set has not been re-normalized or adjusted. As such, direct comparison between the magnitude of the BNT162b2 or mRNA-1273 IgG responses can not be reasonably completed. However, the time dependence of the relaxation of each IgG response relative to their respective peak response can be directly compared. Each IgG fit is therefore normalized by its peak immunity determined through its respective fit. For Fig. 2b–d, the green to red colour sequence distinguishes 25, 50, 75, 99 and >99 IgG% loss relative to peak immunity.We note that the percentage loss milestones are not intended to be relevant immune-correlate markers for vaccine efficacy, rather, they are used as fixed markers to compare humoral loss through time amongst multiple studies. The time at which each percent loss occurs for each vaccine, as well as the humoral degradation rate, $\gamma _{A}$, fit from Eq. (9e), are shown in Table 2.

We find that the humoral degradation rate $\gamma _{A}$ for the two standard dose population fit (which includes both Pfizer and Moderna vaccines) is 0.042 with a relative standard error of 27.2%. The average $\gamma _{A}$ for mRNA-1273 and BNT162b2 is found to be be $0.042\pm 0.003$ and $0.043\pm 0.004\,\hbox {d}^{-1}$, respectively. The time to reach the 25, 50, 75, 99 and >99% loss milestones also varies substantially between vaccine types with 94, 114, 141, and 238 days for mRNA-1273, and 72, 88, 110 and 190 days for BNT162b2. By day 265 following dose one, we find both mRNA-1273 and BNT162b2 are predicted to lead to antibody counts less than $99.5\%$ peak loss.

**Table 2:** Summary of IgG predictions presented.

Vaccine type and dosage	$\gamma A$ ($\hbox {d}^{-1}$)	Time to reach respective percent loss (d)				Loss by day 265 (%)
		25%	50%	75%	99%	
Two standard dose population fit	0.042	83	101	125	212	99.90
Two standard dose BNT162b2	0.043	72	88	110	190	99.96
Two standard dose mRNA-1273	0.042	94	114	141	238	99.67
Single low dose mRNA-1273	0.067	109	134	168	N/A	97.3

Time to reach respective loss values are measured as time since the day of first dose.

In Table 1 we compare our model determined decay rates, and subsequent half-life values, to those found in the literature. Discussion on the values determined by our fits and their agreement with the literature can be found below.

### Long term predictions for two standard doses of mRNA-1273 depending on age

**Figure 3:**
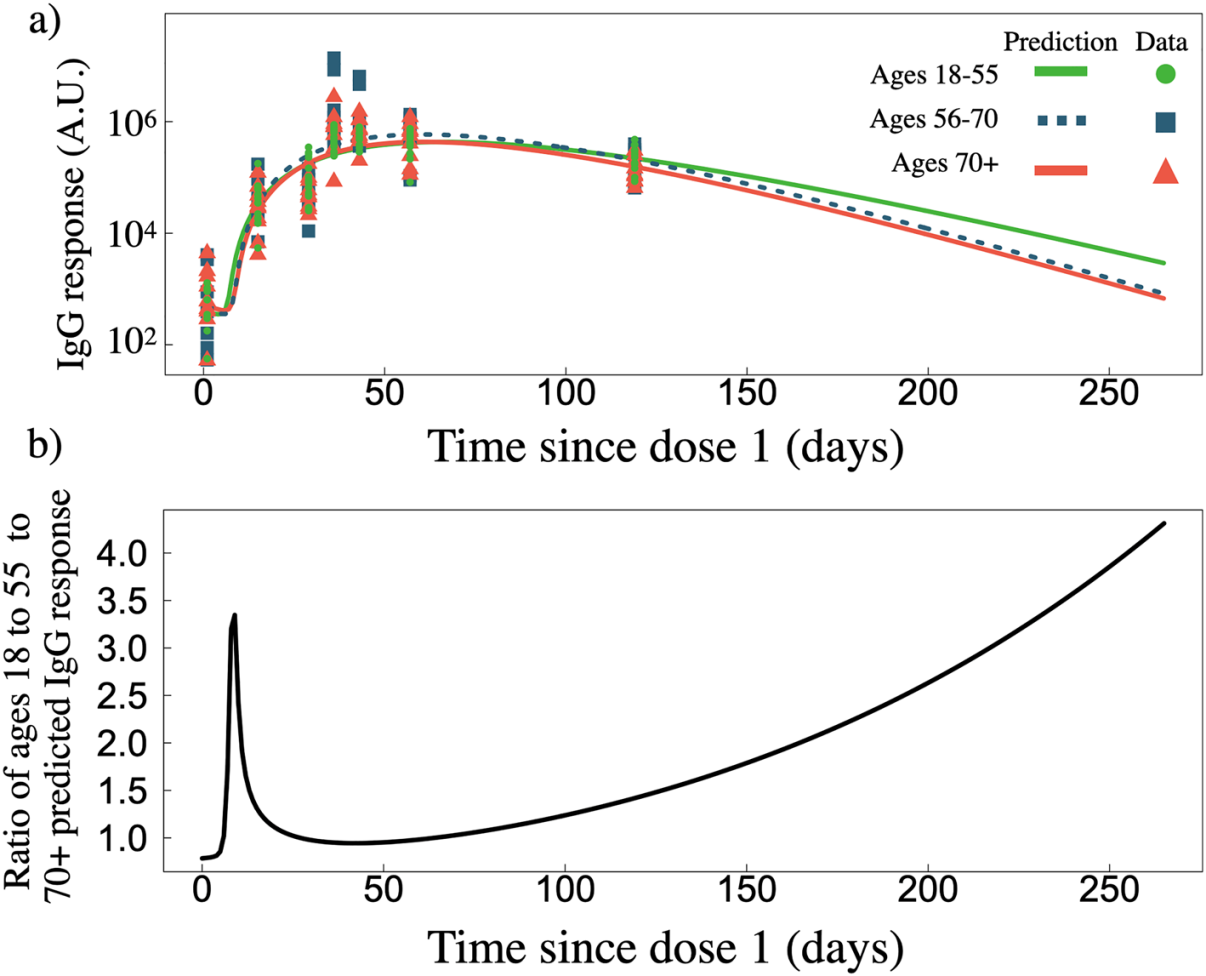
(**a**) IgG response as a function of time since dose one for two standard doses of mRNA-1273, separated by age groups 18–55 ($n = 15$), 56–70 ($n = 9$), and 70+ ($n = 10$) years of age, with data sourced from Ref.^22^. (**b**) Ratio of predicted IgG response of 18–55 age cohort to the 70+ age cohort.

The model fits to data including population delineation by age^22^ allow for analysis of specific study age cohorts. Whereas in Fig. 2a we plot the overall response from all age cohorts, in Fig. 3a we separate the age cohorts into 18–55, 55–70, and 70+ aged individuals and plot the model predicted responses. Reported errors are the standard deviations of the determined fit values. We find nearly identical long-term (post peak) behaviour between the 56–70 and 70+ individuals with $\gamma _{A}$ of $0.045\pm 0.014\,\hbox {d}^{-1}$ and $0.045\pm 0.015\,\hbox {d}^{-1}$, respectively. While for the 18-55 cohort we find $\mu _{BA}$ and $\gamma _{A}$ to be 0.74 and $0.040\pm 0.015\,\hbox {d}^{-1}$, respectively. We find slight differences in the plasma B cell removal rates, $\gamma _{B}$; younger individuals have a $\gamma _{B}$ of $0.05\pm 0.05\,\hbox {d}^{-1}$ while 70+ individuals have a larger $\gamma _{B}$ of $0.06\pm 0.05\,\hbox {d}^{-1}$. Thus, our results suggest little differences in Plasma B cell maintenance across age cohorts, however, antibodies are found to degrade faster in older cohorts. This age-dependent differences is leading to a higher IgG response in younger individuals by day 265 compared to older individuals.

To better illustrate the differences between the predicted responses from 18–55 and 70+ individuals, we compared the ratio of 18-55 and 70+ fitted responses (Fig. 3b). We find that by day $\sim$50 the ratio is $\sim$1, however, as time progresses an advantage for the 18–55 cohort emerges, such that by day 265 the 18–55 age group has roughly four-fold more IgG compared to the 70+ age group. We also find a much stronger initial IgG response in the 18–55 age cohort, where by day ten these individuals have 3 times more IgG, however, this advantage quickly decays. All fitted Eq. (9) model parameters for all age-specific predictions can be found Tables S3 and S4.

### Sex dependant long term predictions for two standard doses of BNT162b2

**Figure 4:**
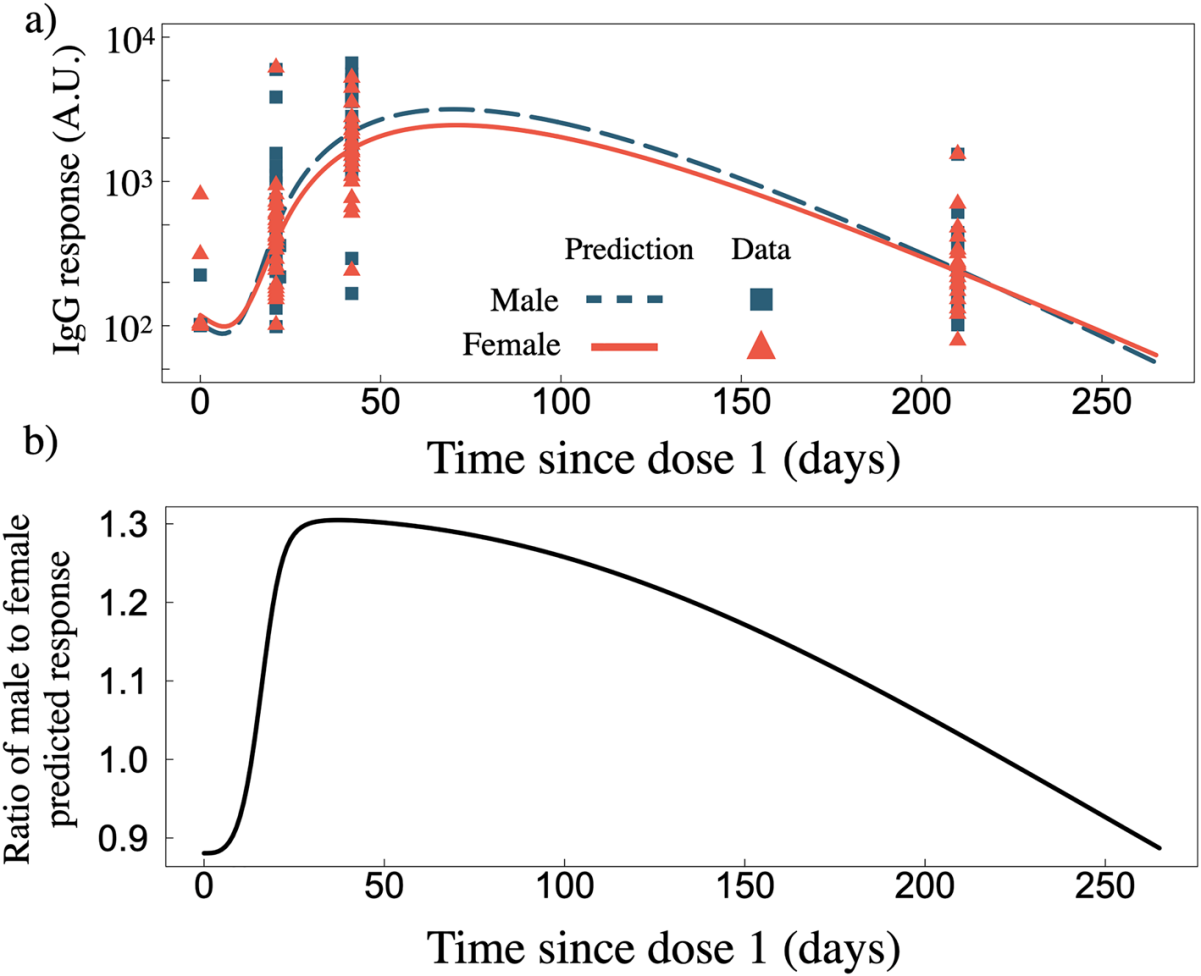
(**a**) IgG response as a function of time since dose one for two standard doses of BNT162b2 for male ($n = 22$) and female ($n = 24$) data sets^24^. (**b**) Ratio of male to female predicted IgG response from panel (**a**).

Amongst the clinical data used for our fit to two standard doses of BNT162b2 is a data set where IgG response is separated by sex^24^. In Fig. 4a we plot the sex-dependent predictions as well as the corresponding clinical data separated by sex. In Fig. 4b we plot the ratio of the male and female predicted response. A higher initial IgG advantage emerges for males that peaks on day 30, however, the immunity advantage slowly dissipates such that by day 265 the ratio of male to female IgG response is $\sim$1.

### Long term predictions for two low doses of mRNA-1273

**Figure 5:**
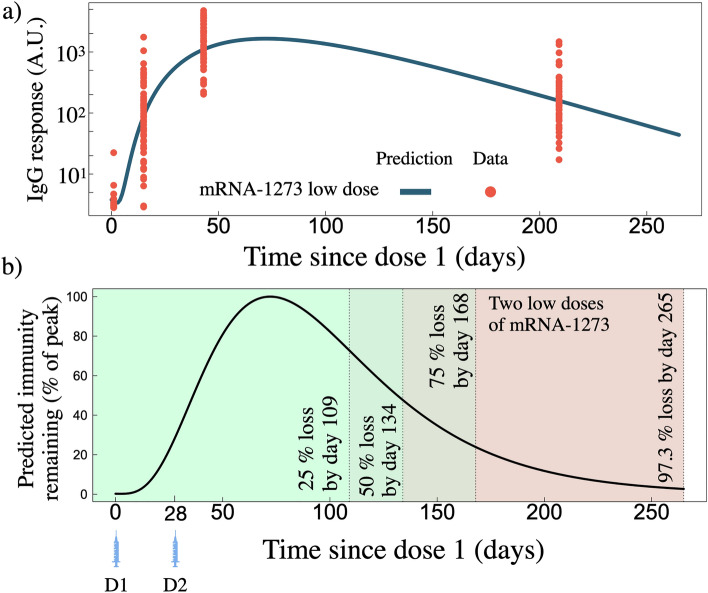
(a) IgG as a function of time since dose one for two low doses of mRNA-1273. Blue line is the model prediction fit from the clinical data ($n = 33$) shown as orange points. (**b**) Predicted IgG remaining, normalized by the peak IgG count, for the two low doses of mRNA-1273 shown in panel (**a**).

Figure 5a displays the IgG population model prediction for two low-doses of mRNA-127 (25 $\upmu$g as opposed to 100 $\upmu$g). The clinical data for these fits was sourced from ref.^25^ (this data set was not included in the standard dose fits described above). Individual fits to the RBD and spike IgG can be found in Fig. S9, all individual fit parameters can be found in Tables S6 and S7, and the two-low-dose population fitted parameters can be found in Table S5. The population fit for two-low-doses mRNA-1273 yields $\gamma _{A} = 0.067 \pm 0.003\,\hbox {d}^{-1}$. The time to reach a percent loss of 25, 50, 75%, relative to peak IgG value is 109, 134, 168 days, and by day 265 we find 97.3% of the peak response has waned (Fig. 5b).

## Discussion

We have developed a novel within-host mathematical model (Eq. 9) to describe the vaccine dynamics from LNP-formulated mRNA vaccines. An in-depth description of our novel model for LNP-based mRNA vaccines is provided in the “Methods” section. We curated data from 8 previously published two-dose mRNA-1273 and BNT162b2 antibody and cytokine clinical trials, comprising a total of 22 data sets (antibody and cytokine measurements). We then utilized the SAEM fitting algorithm in Monolix^26^ (described further in “Parameter estimation, fitting assessment, and long-term simulations” section) to simultaneously fit the data to our model for two standard doses of mRNA-1273^22,23^ and BNT162b2^18–21,23^ (comprising a total of 20 data sets), and also separately fit to 2 datasets for two low doses of mRNA-1273 (obtained from^25^). For all clinical data used in this work, the time between doses is the vaccine developer’s recommended dose interval time. In line with the initial planned dose three interval set out by the CDC^27^, 8 months following dose two, we extend all of our fits to predict the response out to day 265 following dose one.

Table 2 summarizes our long-term predictions shown in Fig. 2a–d. We find humoral immunity gained from two standard doses of mRNA-1273 and BNT162b2 to peak on days 63 and 47 following dose one, respectively. We normalize our curves by the peak IgG value to then determine the milestones for percent loss relative to the peak. A number of modelling studies predicting waning immunity caution SARS-CoV-2 resurgence upon inadequate administration of a booster dose, depending on the severity of how quickly immunity wanes^43–45^. Before the third dose of the vaccine is administered, we predict a significant window of time where individuals will have greater than 99% humoral loss relative to peak immunity of 105 and 27 days for BNT162b2 and mRNA-1273, respectively. Our predictions are therefore in agreement with the CDC’s adjusted timeline of 6 months post dose two for the third dose of the vaccine^28^, as opposed to the initial 8 month post dose two plan^27^. Overall, we find that two doses of mRNA-1273 to decay slower relative to peak immunity compared to two doses of BNT162b2 (Table 2).

The modest humoral advantage as a function of time for mRNA-1273 recipients is in line with the recent CDC MMWR report which found vaccine effectiveness in preventing hospitalization was higher for Moderna recipients compared to Pfizer^46^. Furthermore, individuals who received two-doses of mRNA-1273 have been found to have dramatically reduced neutralizing antibody activity 6 months following dose two^47^; suggesting a 6 month timeline for dose three post dose two^28^.

It is known that statistical power in model data fitting is decreased when study cohort populations are small^23^. We have curated and simultaneously fit 22 datasets of mRNA vaccine clinical trials from previously published studies (see Table 3). The power of the SAEM algorithm employed by Monolix thus provides greater statistical power in the current study. A better perspective on SARS-CoV-2 vaccine-elicited within-host antibody half-lives (and generation and decay of other components of the immune reponse) can then be obtained. Table 1 summarizes the fit-determined decay rates and equivalent half-lives, and compares our fitted values to those found in the literature. We note that the values are mRNA-vaccine specific; we have not analyzed vaccinated recipients who have been challenged by SARS-CoV-2, and we have not analyzed SARS-CoV-2 naive individuals challenged by SARS-CoV-2. We find that the half-life elicited from two doses of BNT16b2 and mRNA-1273 to be 16.1 days and 16.5 days, respectively. The IgG half life values compare well to previously determined SARS-CoV-2 IgG half-life values of 36 days^29^ and 21 days^30^. A study tracking anti-S titres for BNT162b2 vaccines found a significantly longer half life of 68.8 days for BNT162b2^32^, suggesting that other components of the antibody response may have a much longer half-life. However, in the individual fit to the Suthar et al. data set, which tracked antibody waning for 6 months in 56 healthy volunteers who received two doses of the BNT16b2 vaccine, we find the model fit to have good agreement with the data (Fig. S1j,n) and determine a half-life on that fit of 17.8 days. Thus, a fit to data extending out to 6 months post dose 1 reveals results in agreement with antibody waning timescales on the order of $t_{\frac{1}{2}}\sim$20 days, similar to previous reports^29–31^.

A slight decline in Spike+ Memory B cells has been observed for SARS-CoV-2 recovered individuals (Figure 2B from^18^), however, a half-life or decay rate was not provided. We estimate plasma B cells actively producing IgG to have a half-life of $\sim$10 days, however, we stress that this value does not include IgG+ Memory B cells that are maintained within the body and activated upon a SARS-CoV-2 challenge. This predicted decay in antibody-producing Plasma B cells may reflect the fact that upon no SARS-CoV-2 challenge primed plasma B cells may be transitioning to a memory B cell state.

We are unable to find SARS-CoV-2-specific cytokine half lives from the literature. Cytokines are, however, known to have quick in vivo half-lives. Our population fit to the mRNA-vaccine IFN-$\gamma$ data sets from Bergamaschi et al.^20^ and Camara et al.^21^ reveal an IFN-$\gamma$ decay rate ($\gamma _{F}$) of 201.24 d$^{-1}$, corresponding to a half-life of $\sim$5 min. The individual fits, however, show a larger spread in half-life values; Bergamaschi et al.^20^ and Camara et al.^21^ individual-fit-determined half-lives vary between 3.7 and 83 min, respectively, suggesting that significant variability can occur between cytokine data sets containing varied numbers of individuals. Nonetheless, the resulting SARS-CoV-2 vaccine-specific IFN-$\gamma$ half-lives compare well to values determined in studies of both humans and mice (Table 1). We note that a large variability in half-life response is not found for our antibody fits (see Tables S3 and S4).

The novel mathematical model (Eqs. 9a–h) developed in this work represents the complex physiological response elicited by mRNA-based vaccination in humans, from the initial vaccine dosage through to humoral and cellular immune responses. To understand and validate the model, as well as validate the fitted parameter estimates, we performed both structural and practical identifiability analyses. Structural identifiability methods are used to determine whether each model parameter has an effect on the model output^48^.

Since, we focused on state variable peak values and subsequent decay, we performed PRCC sensitivity analysis with LHC sampling on every Eq. (9) parameter and determined the parameter’s influence on variable peak values (the model output), as shown in Figure S13. We found that the vaccine absorption rate $\mu _{LV}$ and decay rate $\gamma _{L}$ (Eq. 9a) had little to no sensitivity on all model outputs, suggesting these parameters may not be identifiable in our fitting approach. The vaccinated cell decay rate, $\gamma _{V}$ (Eq. 9b), represents the rate vaccinated cells cease to influence CD4+ T cell priming; model outputs were found to be highly sensitive to $\gamma _{V}$. Without imposing prior assumptions or boundary conditions on $\gamma _{V}$, we fit this value to be 0.07 $\hbox {d}^{-1}$, which equates to a halflife of $\sim$10 days, demonstrating remarkable consistency with the length of time that mRNA vaccines have been found to actively translate at the site of injection in mice^33^. CD4+ T cell dynamics were also found to have a strong influence on the model output; for this reason, given we are not fitting to CD4+ T cell data specifically, we fixed the CD4+ decay rate to $\gamma _{T}=0.055$ as previously estimated^49^ to avoid structural (and practical) non-identifiable issues. Our sensitivity study unveiled some intuitive influences of parameter values on the model’s outputs. For example, cytokine peak values were determined to be sensitive to their respective cytokine parameters as well as CD8+ T cell dynamics. Likewise, antibody peak values were found to be sensitive to antibody parameters as well as plasma B cell parameters, consistent with physiological truths.

To gain further structural model insight, we also performed a model complexity reduction analysis (supplementary section 6). There, we demonstrated that the model can be decomposed into two distinct phases: the first is a priming phase which represents a cascade of interactions from vaccinated cells through to CD4+ and CD8+ T cells, and the second a complex activator-inhibitor phase between plasma B cells and interleukin. We simulated both the full model and reduced model under the same parameter estimates and uncovered nearly identical solutions (Figure S14).

Practical identifiability attempts to quantify whether the estimated model parameters are well-constrained by the various data sets to which they are being fit^48^. In this vein of practical identifiability, we estimated random effects (REs) via a nonlinear mixed effect model Monolix^26^. This method is particularly useful for interpreting experimental heterogeneity^50^. With respect to two-standard doses, the resulting REs are summarized in Table S2. Following the structural identifiability outcomes described above, we found the dosage dynamics (Eq. 9a) had high random effect values with large relative standard error (RSE), implying parameters in Eq. (9a) have practical non-identifiability, in agreement with our previous sensitivity results. Similarly, the model complexity reduction (supplementary section 6) was found to be robust to parameters $\gamma _L$, $\alpha _{FC}$, and $\alpha _{CF}$ in that the model solutions both with zero and non-zero values were nearly identical (Figure S14). Therefore, non-identifiability in these parameters is likely due to their lack of influence on model structure rather then practical identifiability. Further, we found reliable REs and RSEs on the antibody production and degradation parameters, suggesting our reported antibody dynamics are well-constrained to the data sets used in this work. Generally, for non-fixed parameters where REs were employed in the fitting algorithm, we were able to estimate all random effects, but not always the relative standard error of the random effects (denoted by ‘NaN’ in Table S2). As determined by the model complexity reduction (explored in much further detail in ref.^51^), many of these parameters decouple into quickly-dissipating timescales that are of no practical use to measuring antibody dynamics or cellular immunity. Altogether, these identifiability analyses serve as a strong foundation for employing our model for future data-driven work where the relationship between model parameters and identifiability can be used to derive a more simple well-behaved model structure to determine robust parameter estimates for various serological features beyond those studied in this work.

An immunologic correlate of protection against SARS-CoV-2 has not yet been established^52^. However, previous modelling and statistical studies have considered NAb antibody responses as correlates of protection to estimate vaccine efficacy, where modelled predictions were able to successfully capture efficacies from clinical trials^53^, and furthermore a robust correlation between IgG and NAb has been found between titre response and vaccine efficacy^54^. Our modelling approach allows us to make a clinically-guided qualitative prediction for a humoral-based immunologic correlate of protection. For example, it has been found that 7 months following dose two of Pfizer/Biontech efficacy in preventing infection drops from 75 to 16%^10^. At 7 months past dose two for BNT162b2 we predict IgG levels to drop to 0.16% of the peak response; suggesting that 0.16% of the peak response correlates to 16% efficacy. In another BNT162b2 study, efficacy against infection was found to decline to 47% five months post dose two, with efficacy against the delta variant found to be 53% four months after full vaccination^55^. We find that following two standard doses of BNT162b2 the IgG counts have dropped to 2.1 and 7.2% of peak, 5 and 4 months following the second dose, respectively.

An antibody study of mRNA-1273 found vaccine efficacy as low as 50.8%, and as high as 96.1%, 28 days past dose two, and further found vaccine efficacy to increase throughout day 29–57 past dose one^56^. We find that 28 days past dose two mRNA-1273 IgG levels are predicted to be at 99% of their peak value, and from days 29 to 57 past dose one the IgG levels are predicted to increase towards the peak value, which occurs on day 63 (Fig. 2d).

It is known that as individuals age, they can develop numerous possible molecular immunological impairments that lead to an inability to maintain humoral immunity^57,58^. A decline as a function of age for Ab and B-cell SARS-CoV-2-specific responses from two doses of mRNA vaccines has been previously observed^18^. Therefore, we expanded our study to predict humoral immune loss as a function of age for two-standard-dose mRNA-1273 where data was available to do so. We find that over the first 20 days post dose one, the younger cohort has a much stronger predicted IgG response compared to the 70+ cohort, such that by day 10 they have achieved a three-fold IgG advantage over the older cohort (Fig. 3b). A more rapid antibody response to vaccination is not unexpected in younger individuals, where factors such as impaired B cell development and maintenance are more common in the elderly^59^. Indeed, we find that plasma B cells are produced at roughly the same rate amongst all ages, however, die at a slower rate of 0.044 d$^{-1}$ in younger individuals, compared to a rate of 0.061 d$^{-1}$ in older individuals; which suggests that younger individuals are better at maintaining their plasma B cell population.

The initial humoral advantage in younger individuals quickly dissipates, such that by day 50 the ratio of IgG response between 18–55 and 70+ individuals is $\sim$1.0. As time progresses beyond day 50 after dose one, we find there exists an increased ability to maintain humoral immunity amongst 18-55 aged individuals compared to 56–70 and 70+ aged individuals (Fig. 3b), such that by day 265 the 18–55 aged individuals are predicted to have $\sim$4-fold more IgG compared to older individuals. This result is in line with mRNA-1273 low-dose clinical findings where a two-fold reduction in IgG by day 209 in older cohorts was found^25^, as well as a separate study which found mRNA-1273 and BNT162b2 levels to negatively correlate with age^60^. These results support vaccine administration strategy that prioritizes older individuals^61^, as we predict their humoral immune loss to occur faster compared to younger individuals.

As the world’s nations respond to the continued spread of the SARS-CoV-2 pandemic and the emergence of its many variants, vaccine conservation is becoming increasingly important. As cautioned by the World Health Organization, many individuals in some priority populations have not yet received a primary vaccination^62^. This begs the question; will a lower dose elicit protection against a SARS-CoV-2 challenge? Without an immunologic correlate of protection against SARS-CoV-2 this question is difficult to answer explicitly. However, towards addressing the concern, we fit our model to two-low-dose (25 $\upmu$g) mRNA-1273 data sets (Fig 5), and compared the degradation kinetics relative to peak immunity to our two-standard-dose results (Table 2). All population fit parameters for standard and low dose scenarios are shown side-by-side in Table S2. For the low dose fits we find antibody degradation to be approximately $\sim$1.6 times faster, while the released antibody rate is a factor of $\sim$1.1 times slower, as compared to the standard dose population fits. We find, however, that relative to peak immunity, the two-low-dose strategy loses immunity slower, such that by day 265 since dose one recipients are predicted to have 97.3% loss of peak immunity, compared to $>99$% peak loss for two standard doses of mRNA-1273 or BNT162b2 (Table 2).This result is corroborated by previous studies that demonstrate lower prime dosages (as well as longer separations between dosages) yield a higher efficacy, where the mechanism is hypothesized to be lower doses leading to a selection stringency in germinal centres ultimately leading to B cells with higher affinities for the target antigen^63^. Further vaccine conservation may be realized by spreading the dosage interval beyond the manufacturers initial recommendations. Persistent immugenicity has been found in individuals having received extended prime-dosage-intervals of ChAdOx1 nCoV-19 vaccine^64,65^. We save for future work an exploration of increasing dosage intervals on immunological outcomes from mRNA vaccines using our modelling approach.

Our modelling approach successfully captures humoral immunity gained from vaccination and provides biologically-relevant mechanistic insight into the vaccination immune response. For future work, with appropriately high-resolved data we can expand the model to robustly predict cellular responses, or we can extend the model to include additional booster doses separated by variable timescales. Vaccine-free within-host pathogen dynamics have been studied for SARS-CoV-2^66–72^, coupling a vaccine model, such as that presented in Eq. (9) in this work, with an infection model would be an interesting course of future work to better understand the mechanisms driving immunity vaccination together with challenge by infection.

## Conclusion

In our study we develop a novel within-host mathematical model to describe the vaccination process of LNP-formulated mRNA vaccines, and we fit our model to 22 mRNA-1273 and BNT162b2 clinical data sets to determine best-fit model parameters and establish accurate long term predictions. We separate our individual fits by age, sex, and vaccine type. We find young individuals (18–55 of age) to be more responsive to vaccination as well as maintain humoral immunity longer than compared to older (70+ of age) individuals. Our results suggest males have a slightly higher peak response to two doses of BNT162b2, however, there exists little difference between sexs in the ability to maintain humoral immunity over the long term. We predict that two standard doses of either vaccine results in less than 99% of peak immunity remaining by day 190 and 238 past dose one for BNT162b2 and mRNA-1273, respectively. Relative to peak IgG response, the the mRNA-1273 vaccine is found to decay slower as a function of time as compared to BNT162b2.

Our results will help guide public health policies regarding booster dose timelines. Our humoral results, correlated with efficacy against infection studies, are in agreement with the CDC timeline that a booster of an LNP-formulated mRNA vaccine may be required within 6–8 months of the second dose to maintain high effectiveness against SARS-CoV-2 and the emerging variants^27,28^.

## Methods

### Clinical data acquisition

All clinical data used in this work were previously published and are summarized in Table 3. If unavailable directly from the published source, we digitized the data directly using the software WebPlotDigitizer (version 4.5)^73^. All data sets in the supplementary and main text have been deidentified, and all methods were performed in accordance with the relevant guidelines and regulations.

**Table 3:** Summary of clinical data used in this work.

Reference	Quantities used in this work	Vaccine	Dosage	Population size
^18^	RBD IgG, Spike IgG	BNT162b2 &mRNA-1273	2 dose, 30 $\upmu$g or 100 $\upmu$g	33 (32 BNT162b2 & 1 mRNA-1273)
^19^	Spike IgG	BNT162b2	2 dose, 30 $\upmu$g	124 for dose 1 69 for dose 2
^20^	Spike-RBD IgG, IFN-$\gamma$,IL-6, IL-8, IL-15, IL-16	BNT162b2	2 dose, 30 $\upmu$g	63
^21^	Spike IgG, IFN-$\gamma$	BNT162b2	2 dose, 30 $\upmu$g	20
^22^	RBD IgG	mRNA-1273	2 dose, 100 $\upmu$g	34
^25^	Spike IgG, RBD IgG	mRNA-1273	2 dose, 25 $\upmu$g	33
^23^	Spike IgG, RBD IgG	BNT162b2 &mRNA-1273	2 dose, 30 $\upmu$g or 100 $\upmu$g	20 (6 BNT162b2 & 14 mRNA-1273)
^24^	Spike IgG	BNT162b2	2 dose, 30 $\upmu$g	46 (24 females, 22 males)

Some publications listed studied in-host vaccine dynamics from both Sars-CoV-2 naive and previously infected indivduals, in this work we only digitized those data that are in the naive category and report the associated population size.

### Model for in-host mRNA vaccination

Here we describe our model for mRNA vaccination delivered by lipid nano particles, and the subsequent in-host immunization process. We model the time dependence of eight state variables: lipid nanoparticles (*L*), vaccinated cells (*V*), CD4$^{+}$ cells (*T*), plasma B cells (*B*), antibody (*A*), CD8$^{+}$ cells (*C*), and the cytokines interferon (*F*) and interleukin (*I*). The model developed in this work is adapted from our recently published adenovirus-based model^40^.

#### Inoculation and vaccination

BNT162b2 and mRNA-1273 both contain base-modified—or nucleoside-modified—*bm*RNA that encode for full-length diproline-stabilized SARS-COV-2 viral spike protein, and are delivered via a payload enclosed by a lipid nanoparticle (LNP)^74,75^. The standard mRNA dose in BNT162b2 is 30 $\upmu$g, and together with the known mRNA size of 4.3 kb^74^ and average nucleotide molecular weight of 319 g  mol, there are an estimated $1.32\times 10^{13}$ of *bm*RNA in each dose. The standard dosage of mRNA-1273 contains 100 $\upmu$g of *bm*RNA, while the molecular weight of the molecule is not public knowledge. LNP-based mRNA theraputics are a novel techonology where the pathway to activating the innate and and adpative immune response in humans is not well understood. Further, the route of administration of an LNP-based vaccine has a pronouced effect on the targeted cell types and tissues; for example, a luciferase-based mouse study by Pardi et al. found that intradermally, intraperitoneally, subcutaneously, intramuscularly, and intravenously inocculated LNP vaccines have varying outcomes^33^. While intraperitoneally and intravenously delivered LNP vaccines tended to primarily drain to hepatocytes in the liver, intramuscularly, subcutaneously, and intradermally delivered LNP vaccine stayed near the site of injection for many days (with partial drainage to the liver). A later study examining intramuscular injection in mice found that an LNP-based mRNA vaccine activated the innate immune system and remained active near the injection site as well as in draining lymph nodes^76^. The cytokines TNF and IL-6 were found in the muscle as as well as in the draining lymph nodes, as well as activation of B cells, and CD8$+$, CD4$+$ T cells. Motivated by such studies, the SARS-CoV-2 mRNA-based vaccines BNT162b2 or mRNA-1273 are delivered intramuscularly to humans.

Upon receiving a dosage of BNT162b2 or mRNA-1273, the patient is therefore likely producing spike protein near the site of injection for many days, as well as in lymph nodes for a period of time. The timescales and activation thresholds between the innate and adaptive immune response, driven by the proportions of the LNP dose absorbed into myocytes, draining lymph nodes, and hepatocytes is not well known. Therefore, in this work, we take a course grained approach to the in-host immunization process and model a general ‘vaccinated’ cell compartment.

The mechanistic model we propose for innoculation and preceding immunization in-host from an LNP-based mRNA vaccine is as follows. The number of LNPs in-host after inoculation is

1 $$\begin{aligned} \frac{d\textrm{L}}{dt} = - \mu _{L,V}{\textrm{L}} - \gamma _{\textrm{L}}\textrm{L}. \end{aligned}$$

The $\mu _{L,V}$ term accounts for the interaction between the LNP-mRNA payload and target cell, *V*. We consider a cell successfully vaccinated upon the successful fusion of a LNP, leading to subsequent expression of the spike protein (which is not explicitly modelled). $\gamma _{\textrm{L}}\textrm{L}$ captures the natural degradation of LNPs in-host, as well as fusion inefficiency and drainage of the LNPs to the liver. The vaccinated cell count, describing cells capable of producing antigen, is then

2 $$\begin{aligned} \frac{d\textrm{V}}{dt} = \mu _{L,V}\textrm{L} - \gamma _{V}V, \end{aligned}$$

where $\gamma _{V}$ is a general decay rate describing the loss of ability of the cell to produce antigen (not necessarily through cell death). For example, intramuscularly delivered LNP-mRNA in mice has been shown to translate locally at the cite of injection for up to 10 days^33^; a decline in translation may be caused by a number of cytoplasmic enzymes^77^. In this work we do not impose any boundaries on $\gamma _{V}$.

#### CD4$^{+}$ cell priming by vaccinated cells

Having received the mRNA payload vaccinated cells express antigen, leading to the development of membrane-bound class-II peptide-major histocompatibility complexes (MHC-II). Naive CD4$^{+}$ T-helper cells (given by *T* in our model) recognize and bond with MHC-II where an information exchange occurs^78^. These dynamics are captured by the $\mu _{T,V}$ term in our model. The CD4$^{+}$ cell is then considered primed with antigen information. We therefore model the activation of naive CD4$^{+}$ cells by

3 $$\begin{aligned} \frac{dT}{dt} = \mu _{T,V}V - \gamma _{T}T, \end{aligned}$$

where $\mu _{T,V}$ is the rate of interaction between naive *T* cells and vaccinated cells and $\gamma _{T}$ is the natural death rate of *T* cells. In this work $\mu _{T,V}$ is determined through a fit to clinical data. The decay rate is fixed to 0.055 d$^{-1}$ as determined in a previous study^49^.

#### Humoral immunity

We model a primary antibody response generated by plasma B cells (*B*). Plasma B cells are primed by antigen-specific T cells^79^. In our model this is taken into account by the interaction rate $\mu _{TB}$. The maturation of a naive B cell into an antibody secreting cells is further enhanced by various interleukin^79,80^. We incorporate interleukin-mediated plasma B cell maturation into our model through the term $\alpha _{_{BI}}\left( \frac{I}{S_{_{I}} + I}\right)$. Thus the term $\alpha _{_{BI}}$ regulates Plasma B cell stimulation by interleukin, while $S_{_{I}}$ provides a duplication threshold of plasma B cells due to interleukin. We fix $s_{_{I}} = 1000$, which was determined by the two-dose half-max interleukin threshold in our previous work on adenovirus vaccines^40^, and is further justified as accurate for this current work by the maximum clinical IL-8 values determined from the BNT162b2 interleukin data set obtained from ref.^20^. In this work we use four interleukin data sets: IL-6, IL-8, IL-15, and IL-16. Previous studies have linked all four of these interleukins to Plasma B cell differentiation or proliferation^81–86^, thus we incorporate all four of these interleukin species into our fits. We therefore model the production of antigen-specific plasma B cells, *B*, as

4 $$\begin{aligned} \frac{dB}{dt} = \mu _{T,B}T + \alpha _{_{BI}}\left( \frac{I}{s_{_{I}} + I}\right) B - \gamma _{B}B, \end{aligned}$$

where $\gamma _{B}$ is the natural death rate of plasma *B* cells. The production of interleukin (I) by CD4$^{+}$ cells is described in “Model for in-host mRNA vaccination” section.

In this work we focus on the production of the predominant antibody in humans immunoglobulin G (IgG)^87^, and do not distinguish between subclasses of IgG or consider other types of immunoglobulin. In our model IgG is given by *A*, and we model the production of *A* by *B* through the source term $\mu _{B, A}B$. The rate of change of *A* is then

5 $$\begin{aligned} \frac{dA}{dt} = \mu _{_{BA}}B - \gamma _{A}A, \end{aligned}$$

where $\gamma _{A}$ is the antibody degradation term.

#### CD8$^{+}$ cell priming and cytokine response

CD8$^{+}$ Cyotoxic T cells, denoted by *C* in our model, target and eliminate virus-infected cells. CD8$^{+}$ cells are primed by antigen presenting cells (Eq. 2) through cell-surface bound MHC class I molecules^88^. In our model this process is captured by the source term $\mu _{CV}V$. The regulation of antigen-specific CD8$^{+}$ by cytokines is complex. Type I IFN has been shown to enhance the CD8+ T cell response during priming^89^, and Type II IFN (IFN-$\gamma$) has been directly shown to enhance development of CD8+ memory^90,91^. We therefore model the enhancement of CD8+ memory development through a source term dependent on the presence of IFN-$\gamma$ (denoted by *F* in our model).

The rate of change of CD8$^{+}$, *C*, cells is then

6 $$\begin{aligned} \frac{dC}{dt} = \mu _{CV}V + \alpha _{C,F}\left( \frac{F}{S_{F} + F}\right) C - \gamma _{C}C, \end{aligned}$$

where $\gamma _{C}$ is the natural death rate of CD8$^{+}$ cells, $\alpha _{C,F}$ accounts for stimulation by *F*, and $S_{_{F}}$ is the duplication threshold due to *F*. In this work we fix $s_{_{F}} = 600$ which was determined by the two-dose half-max IFN-$\gamma$ threshold in our previous work on adenovirus vaccines^40^, and is further justified as accurate based on the two-dose IFN-$\gamma$ data used in this work from refs.^21^ and^20^. The CD8$^{+}$ decay rate is fixed to 0.01 d$^{-1}$ as determined in a previous study^92^.

CD4$^{+}$ and CD8$^{+}$ cells exhibit complex cytokine secretion and regulation dynamics. Recent studies on SARS-CoV-2 infection in humans have shown that cytokines are primarily produced by CD4+ cells, and that CD8+ are suppressed throughout the course of infection^12,93^. Here, we consider a simplified approach where IFN-$\gamma$ and Interleukin (*I*) cytokine production by primed CD4$^{+}$ cells. CD4$^{+}$ cells are one the predominant sources of cytokine production^94^; CD4+ cells have been shown to secrete IFN-$\gamma$^95,96^ and are one of the primary synthesizers of interleukins^86,97^. We therefore model the production of IFN-$\gamma$ (*F*) and interleukin (*I*) by

7 $$\begin{aligned} \frac{dF}{dt} = \mu _{_{TF}}T - \alpha _{_{FC}}CF - \gamma _{F}F, \end{aligned}$$

and

8 $$\begin{aligned} \frac{dI}{dt} = \mu _{_{TI}}T -\alpha _{_{IB}}IB - \gamma _{I}I. \end{aligned}$$

The clearance of *F* and *I* is described by $\gamma _{F}$, and $\gamma _{I}$, respectively. Upon CD8+ priming enhancement by *F*, we model the subsequent removal of *F* by $\alpha _{_{FC}}CF$. Similarly, upon Plasma B cell priming enhancement by *I*, we model the subsequent removal of *I* by $\alpha _{_{IB}}IB$; thus, we do not allow *F* and *I* to influence the rate of priming of multiple cells.

The complete model we use to describe the in-host immunization process by LNP-formulated mRNA vaccines is shown schematically in Fig. 1 and is given by

9a $$\begin{aligned} \frac{d\textrm{L}}{dt}&= - \mu _{_{LV}}{\textrm{L}} - \gamma _{\textrm{L}}\textrm{L} \end{aligned}$$

9b $$\begin{aligned} \frac{d\textrm{V}}{dt}&= \mu _{_{LV}}\textrm{L} - \gamma _{_{V}}V \end{aligned}$$

9c $$\begin{aligned} \frac{dT}{dt}&= \mu _{_{TV}}V - \gamma _{_{T}}T \end{aligned}$$

9d $$\begin{aligned} \frac{dB}{dt}&= \mu _{_{TB}}T + \alpha _{_{BI}}\left( \frac{I}{s_{I} + I}\right) B - \gamma _{_{B}}B \end{aligned}$$

9e $$\begin{aligned} \frac{dA}{dt}&= \mu _{_{BA}}B - \gamma _{_{A}}A \end{aligned}$$

9f $$\begin{aligned} \frac{dC}{dt}&= \mu _{_{CV}}V + \alpha _{_{CF}}\left( \frac{F}{s_{F} + F}\right) C - \gamma _{_{C}}C \end{aligned}$$

9g $$\begin{aligned} \frac{dF}{dt}&= \mu _{_{TF}}T - \alpha _{_{FC}}CF - \gamma _{_{F}}F \end{aligned}$$

9h $$\begin{aligned} \frac{dI}{dt}&= \mu _{_{TI}}T -\alpha _{_{IB}}IB - \gamma _{_{I}}I. \end{aligned}$$

Our model appears to be a complex 8 equation coupled model, however, demonstrated analytically in supplementary section 6, the model readily decouples and reduces to a more simple activation-inhibition model between plasma B-cells and interleukin. We save for future work a full analytical exploration of the multiscaledness and complexity reduction^51^.

### Parameter estimation, fitting assessment, and long-term simulations

All fits to clinical data using our model (Eq. 9) were performed in Monolix^26^ (Version 2020R1) using non-linear mixed-effects models. IgG and IFN-$\gamma$ concentrations vary over many orders of magnitude over time, as such we log-transformed these quantities during fitting, while we fit to the linear interleukin response. Each data set used in this work is listed in Table 3; ‘individual study fits’ refers to aggregated data from the entire population of individuals within each respective study listed in Table 3. Individual study fit parameters (Tables S3 and S4) for each data set are determined by the maximum likelihood estimator Stochastic Approximation Expectation-Maximization (SAEM), and all fits met the standard convergence criteria (complete likelihood estimator). For IgG data sets from Ref.^23^, which begin on day $\sim$50 past dose one, an interval-censored data point was added to day 1 to guide the initial condition fit, where the allowable initial range of the fit is bounded within all the initial day 1 IgG values used in this work. Final clinical data points for IL-15 and IL-6 were gathered immediately after dose two where these quantities have peaked, such that the subsequent decay was not characterized^20^. To ensure the eventual decrease in these cytokines as a function of time in our modelled prediction, we added an interval-censored data point on day 200, which ensures the fit on this day is between 0 and the minimal value determined on day 1 for each respective concentration. All two-standard-dose data sets are fit simultaneously in Monolix yielding a single standard dose population fit as well as individual fit parameters corresponding to each standard-dose data set. The two-standard-dose BNT162b2 and mRNA-1273 results are then determined by averaging the individual fit results determined from their respective vaccine type; thus, for example, our final mRNA-1273 model prediction takes into account all mRNA-1273 clinical data sets used in this work. Similarly, all two-low-dose data sits are fit simultaneously in Monolix yielding a single low dose population fit as well as individual fit parameters corresponding to each low-dose data set. For all data sets we fit to cohorts of individuals, that is, the data were grouped and fit for each study, but not for each individual person within a study.

### Sensitivity analysis

We perform sensitivity analysis to characterize the response of our model outputs to variation in the fitted parameters^98^. Latin hypercube sampling (LHC) and partial rank correlation coefficient (PRCC)^99^ are employed to study the effects of model outcomes on the peak value of each state variable. We focus our sensitivity analysis on the peak values as the peak humoral response have been shown to be predictors of immune protection from symptomatic SARS-CoV-2 infection^100^. For a particular model paramter, PRCC values close to the maximum value of 1.0 indicate model output is highly sensitive to variation in that parameter, with values greater than 0.5 considered significant^101^. PRCC values are either negatively or positively correlated with the model outcome (peak response)^102^.

## Supplementary Information

Supplementary Information.

## Data Availability

All data that support the findings of this study are available within the manuscript and the supplementary information files or from the corresponding authors upon reasonable request.
