# Supplementary material for: Long-term durability of immune responses to the BNT162b2 and mRNA-1273 vaccines based on dosage, age and sex
Source: Sci Rep. 2022 Dec 8;12:21232. doi: 10.1038/s41598-022-25134-0 (PMC9732004; doi:10.1038/s41598-022-25134-0)
Supplement: Supplementary file 1 — Supplementary Information. [file 41598_2022_25134_MOESM1_ESM.pdf]

Long-term durability of immune responses to the BNT162b2 and mRNA-1273 vaccines based on dosage, age and sex

November 1, 2022

Chapin S. Korosec, Suzan Farhang Sardroodi, David W. Dick, Samaneh Gholami, Mohammad Sajjad Ghaemi, Iain R. Moyles, Morgan Craig, Hsu Kiang Ooi, and Jane M. Heffernan

Corresponding author emails:

chapinSkorosec@gmail.com

jmheffer@yorku.ca

Contents

1 Supporting Text **3**

2 Population fit values for two standard doses **3**

3 Two standard doses mRNA vaccination of BNT162b2 or mRNA-1273 **3**

3.1 Individual data set fitted values 5

3.2 Individual fits to IgG data sets 7

3.3 Individual fits to IFN-γ data sets 8

3.4 Individual fits to Interleukin data sets 10

3.5 Goodness of fit predictive checks and parameter distributions 11

3.6 Standardized random effects 13

4 Two low doses of mRNA-1273 vaccination **14**

4.1 Model parameter population fits and individual data set fitted values 16

4.2 Individual fits to IgG data sets 16

4.3 Goodness of fit predictive checks 17

4.4 Standardized random effects 18

5 Sensitivity Analysis **21**

6 Model complexity reduction **22**

1 Supporting Text

Variable	Definition	Units	Initial condition
L	Lipid nano particle	A.U.	1
V	Vaccinated cell	A.U.	0
T	CD4+ T cell	A.U.	0
B	Plasma B cell	A.U.	0
A	Antibodies	A.U	A_0
C	CD8+ T cell	A.U.	0
F	IFN-γ	pg/ml	0
I	Interleukin	pg/ml	I_0

Table S1: Model variables and initial conditions for individual and population fits used throughout this work. We simultaneously fit to multiple IgG data sets, where the data sets come from different labs and have arbitrary units (a.u.). We therefore fit an initial condition A given by A_0. We furthermore simultaneously fit to 4 different interleukin data sets, each having varying responses through time and initial dynamics. We therefore fit the initial condition I_0. The initial conditions in this table represent the *population* initial condition from our fits; every individually-fitted data set may have slightly varying initial dynamics.

2 Population fit values for two standard doses

The population fit values are determined through a fit to all individual data data sets use for each vaccine dosage regimen. Values and figures for every individual fit for each data set are shown in the following sections.

3 Two standard doses mRNA vaccination of BNT162b2 or mRNA-1273

All individual fits shown in this section were fit simultaneously in Monolix.

		Population fit values		
Parameter	Definition	Two standard doses (RSE %)	SD Random Effects (RSE %)	Comment
μ_{LV}	LNP absorption rate with antigen presenting cells	0.91 (NaN)	1.41 (71)	Fit
γ_L	LNP degradation rate	0.00013 (NaN)	2.87 (260)	Fit
γ_V	Antigen presenting cell death rate	0.07 (83.3)	0.5 (40)	Fit
μ_{TV}	CD4+ activation rate by vaccinated cells	4.98 (228)	1.31 (26)	Fit
γ_T	CD4+ natural death rate	0.055 (NA)	0.54 (NA)	Ref. [1]
μ_{TB}	Plasma B cell activation rate by CD4+ cells	0.098 (29)	0.8 (NaN)	Fit
α_{BI}	Plasma B cell stimulation by Interleukin	2.4 (21)	0.45	Fit
S_I	Plasma B cell duplication threshold due to Interleukin	1000 (NA)	(NA)	Fixed
γ_B	Plasma B cell natural death rate	0.071 (49)	0.91 (107)	Fit
μ_{BA}	Released antibody rate by plasma B cells	0.51 (26.9)	0.98	Fit
γ_A	Antibody natural degradation rate	0.042 (27.2)	0.37 (40.2)	Fit
μ_{CV}	CD8+ activation rate by vaccinated cells	0.000022 (65)	3.93 (300)	Fit
α_{CF}	CD8+ stimulation by IFN-γ	0.0000014 (NaN)	NA	Fit (no REs)
S_F	CD8+ duplication threshold due to IFN-γ	600 (NA)	NA	Fixed
γ_C	CD8+ natural death rate	0.01 (NA)	5.1 (NA)	Ref. [2]
μ_{TF}	IFN-γ stimulation rate by Thelper cells	194.79 (191)	0.6 (NaN)	Fit
α_{FC}	IFN-γ clearance by cytotoxic Tcells	0.0000013 (NaN)	NA	Fit (no REs)
γ_F	IFN-γ natural degradation rate	201.24 (179)	1.78 (NaN)	Fit
μ_{TI}	Interleukin secretion by CD4+ cells	2.07 (102)	1.7 (NaN)	Fit
α_{IB}	Interleukin clearance by Plasma B cells	0.0019 (222)	0.64 (NaN)	Fit
γ_I	Natural interleukin degradation rate	0.027 (179)	0.76 (NaN)	Fit
A_0	Antibody initial condition	22.5 (75)	1.2 (21)	Fit
I_0	Interleukin initial condition	5.18 (41)	1.9 (68)	Fit
BIC	Bayesian Information Criteria	3468		Fit
AIC	Akaike Information Criteria	3421		Fit

Table S2: Model parameters definition and population fitted values for two standard doses of BNT162b2 or mRNA-1273. The dosing times are separated by 21 and 28 days for BNT162b2 and mRNA-1273, respectively. A value of “NA” refers to “Not Applicable”, whereby the random effects were turned off for said parameter because it was fixed for all individual study fits or the parameter was fit but due to clear identifiability issues (explored in the Model complexity reduction section below) random effects were turned off. A value of “NaN” means our algorithm was unable to estimate the Relative Standard Error (RSE) for that parameter.

3.1 Individual data set fitted values

Data set ID	Figure reference, quantity used	Vaccine	μ_{LV}	γ_L	γ_V	μ_{TV}	γ_T	μ_{TB}	α_{BI}	S_I	γ_B	μ_{BA}	γ_A
Goel at al. [3]	Fig. 1b. RBD IgG	BNT162b2 & mRNA-1273	0.77	0.00012	0.07	0.75	0.054	0.07	2.67	1000	0.18	0.27	0.042
Goel et al. [3]	Fig. 1b, Spike IgG	BNT162b2 & mRNA-1273	1.08	0.00012	0.07	1.34	0.055	0.077	2.42	1000	0.12	0.5	0.043
Stankov et al. [4]	Fig. 1a, Spike IgG	BNT162b2	0.86	0.0001	0.081	9.42	0.068	0.092	2.84	1000	0.31	0.07	0.048
Bergamaschi et al. [5]	Fig. 1a, Spike-RBD IgG,	BNT162b2	1.08	0.00013	0.079	1.97	0.065	0.078	2.32	1000	0.21	0.91	0.047
Camara et al. [6]	Fig. 1b, Spike IgG,	BNT162b2	0.94	0.00013	0.083	4.05	0.067	0.082	2.63	1000	0.21	0.22	0.048
Bergamaschi et al. [5]	Fig. 2A, IFN-γ	BNT162b2	3.88	0.00012	0.067	4.02	0.051	0.098	2.41	1000	0.071	0.51	0.042
Camara et al. [6]	Fig. 1a, IFN-γ,	BNT162b2	1.14	0.00012	0.057	8.21	0.044	0.098	2.39	1000	0.071	0.5	0.042
Bergamaschi et al. [5]	Fig. 2c, IL-6	BNT162b2	0.36	0.00011	0.071	0.81	0.059	0.094	2.39	1000	0.073	0.53	0.042
Bergamaschi et al. [5]	Fig. 2b, IL-8	BNT162b2	1.04	0.00012	0.13	7.16	0.11	0.088	4.44	1000	0.026	0.45	0.042
Bergamaschi et al. [5]	Fig. 2a, IL-15	BNT162b2	0.45	0.00012	0.068	0.78	0.054	0.096	2.39	1000	0.074	0.53	0.042
Widge et al. [7]	Fig. 1a (RBD antibody, 18-55 yrs)	mRNA-1273	0.9	0.00012	0.06	12.44	0.048	0.096	2.43	1000	0.044	0.74	0.04
Widge et al. [7]	Fig. 1a (RBD antibody, 56-70 yrs)	mRNA-1273	0.17	0.00011	0.066	14.55	0.057	0.083	2.13	1000	0.062	0.94	0.045
Widge et al. [7]	Fig. 1a (RBD antibody, 70+ yrs)	mRNA-1273	0.17	0.00013	0.066	12.73	0.056	0.1	2.25	1000	0.061	0.87	0.045
Bergamaschi et al. [5]	Fig. 2b (IL-16)	BNT162b2	1.64	0.00012	0.067	26.84	0.053	0.11	2.2	1000	0.54	0.54	0.042
Wang et al. [8]	Fig. 1e (RBD IgG)	mRNA-1273	0.97	0.00012	0.064	6.36	0.049	0.098	2.45	1000	0.044	0.56	0.039
Wang et al. [8]	Fig. 1f (Spike IgG)	mRNA-1273	0.95	0.00012	0.064	6.57	0.048	0.099	2.45	1000	0.043	0.57	0.039
Wang et al. [8]	Fig. 1e (RBD IgG)	BNT162b2	0.93	0.00012	0.066	7.25	0.052	0.099	2.47	1000	0.057	0.59	0.04
Wang et al. [8]	Fig. 1f (Spike IgG)	BNT162b2	1.05	0.00012	0.065	7.2	0.05	0.098	2.47	1000	0.053	0.6	0.04
Suthar et al. [9]	Fig. 1a (Spike IgG, Male)	BNT162b2	1.2	0.00012	0.068	6.57	0.051	0.11	2.36	1000	0.03	0.38	0.039
Suthar et al. [9]	Fig. 1a (Spike IgG, Female)	BNT162b2	0.99	0.00012	0.072	5.23	0.054	0.13	2.2	1000	0.026	0.42	0.039

Table S3: Individual fit values for to the various data sets used in this work. This table contains all fitted parameters from equations 9a, 9b, 9c, 9d, and 9e.

Data set ID	Paper Fig. reference, quantity used	Vaccine	μ_{CV}	α_{CF}	S_F	γ_C	μ_{TF}	α_{FC}	γ_F	μ_{TI}	α_{IB}	γ_I	A_0	I_0
Goel at al. [3]	Fig. 1b, RBD IgG	BNT162b2 & mRNA-1273	0.000021	1.4E-06	600	0.0078	194.83	1.3E-06	202.39	2.97	0.0019	0.022	0.58	2.14
Goel et al. [3]	Fig. 1b, Spike IgG	BNT162b2 & mRNA-1273	0.000022	1.4E-06	600	0.014	195.27	1.3E-06	199.53	1.14	0.0016	0.024	0.52	3.74
Stankov et al. [4]	Fig. 1a, Spike IgG	BNT162b2	0.000024	1.4E-06	600	0.018	185.85	1.3E-06	196.98	1.33	0.0035	0.026	1.12	3.23
Bergamaschi et al. [5]	Fig. 1a, Spike-RBD IgG	BNT162b2	0.00002	1.4E-06	600	0.0083	193.89	1.3E-06	207.77	2.34	0.0013	0.024	3.69	3.54
Camara et al. [6]	Fig. 1b, Spike IgG	BNT162b2	0.000022	1.4E-06	600	0.0028	195.21	1.3E-06	202.22	1.35	0.0023	0.026	2.88	3.52
Bergamaschi et al. [5]	Fig. 2A, IFN-γ	BNT162b2	0.000022	1.4E-06	600	0.0094	189.29	1.3E-06	266.97	2.27	0.0019	0.027	22.85	5.34
Camara et al. [6]	Fig. 1a, IFN-γ	BNT162b2	0.000022	1.4E-06	600	0.01	207.17	1.3E-06	124.78	2.07	0.0019	0.027	23.4	4.96
Bergamaschi et al. [5]	Fig. 2c, IL-6	BNT162b2	0.000024	1.4E-06	600	0.0093	191.21	1.3E-06	230.1	0.035	0.0018	0.05	18.39	0.89
Bergamaschi et al. [5]	Fig. 2b, IL-8	BNT162b2	0.00002	1.4E-06	600	0.011	193.87	1.3E-06	212.17	12.28	0.0017	0.027	26.26	0.65
Bergamaschi et al. [5]	Fig. 2a, IL-15	BNT162b2	0.000022	1.4E-06	600	0.011	192.95	1.3E-06	200.71	0.049	0.0018	0.035	23.43	2.06
Widge et al. [7]	Fig. 1a (RBD antibody, 18-55 yrs)	mRNA-1273	0.000022	1.4E-06	600	0.01	195.09	1.3E-06	200.28	15.79	0.0016	0.028	404.79	4.95
Widge et al. [7]	Fig. 1a (RBD antibody, 56-70 yrs)	mRNA-1273	0.000021	1.4E-06	600	0.004	194.37	1.3E-06	224.13	25.77	0.0014	0.028	440.37	5.18
Widge et al. [7]	Fig. 1a (RBD antibody, 70+ yrs)	mRNA-1273	0.000026	1.4E-06	600	0.02	192.49	1.3E-06	195.36	22.97	0.0015	0.028	515.75	3.44
Bergamaschi et al. [5]	Fig. 2b (IL-16)	BNT162b2	0.000022	1.4E-06	600	0.0089	195.54	1.3E-06	203.12	13.22	0.0021	0.027	21.99	235.56
Wang et al. [8]	Fig. 1e (RBD IgG)	mRNA-1273	0.000022	1.4E-06	600	0.009	194.74	1.3E-06	201.76	3.52	0.0018	0.027	50.9	5.21
Wang et al. [8]	Fig. 1f (Spike IgG)	mRNA-1273	0.000022	1.4E-06	600	0.01	195	1.3E-06	200.52	3.45	0.0018	0.027	50.82	5.13
Wang et al. [8]	Fig. 1e (RBD IgG)	BNT162b2	0.000022	1.4E-06	600	0.01	194.79	1.3E-06	201.13	4.09	0.0018	0.027	50.87	5.16
Wang et al. [8]	Fig. 1f (spike IgG)	BNT162b2	0.000021	1.4E-06	600	0.01	195.24	1.3E-06	199.48	4.1	0.0018	0.027	51.08	5.33
Suthar et al. [9]	Fig. 1a (Spike IgG, Male)	BNT162b2	0.000021	1.4E-06	600	0.012	195.2	1.3E-06	200.65	0.43	0.0022	0.028	104.2	6.21
Suthar et al. [9]	Fig. 1a (Spike IgG, Female)	BNT162b2	0.000022	1.4E-06	600	0.012	195.21	1.3E-06	201.71	0.43	0.0025	0.028	118.33	7.31

Table S4: Individual fit values for to the various data sets used in this work. This table contains all fitted parameters from equations 9f, 9g and 9h.

3.2 Individual fits to IgG data sets

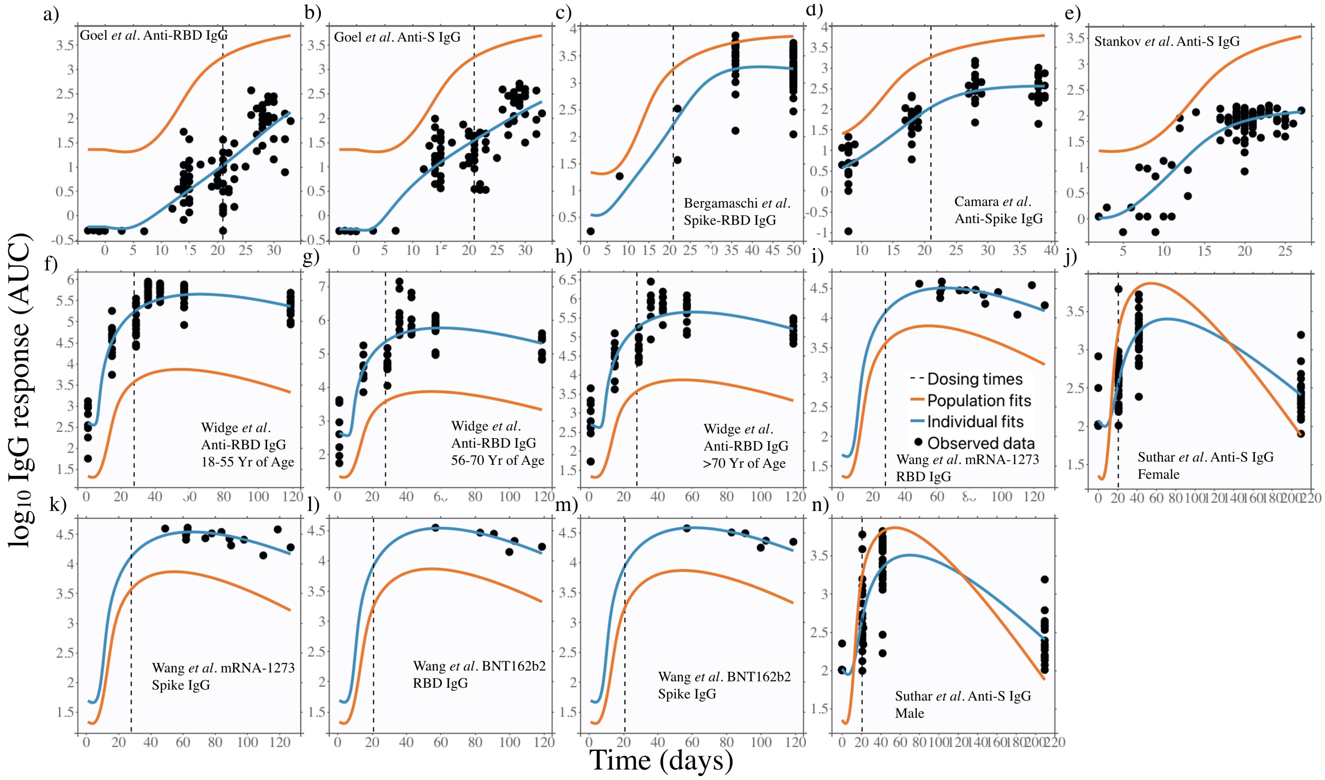

Figure S1: Individual fits to all standard dose IgG data sets used in this work. References for the data set sources can be found in Table 1 of the main text, all individual fitted parameters for each fit can be found in Tables S3 and S4.

3.3 Individual fits to IFN-γ data sets

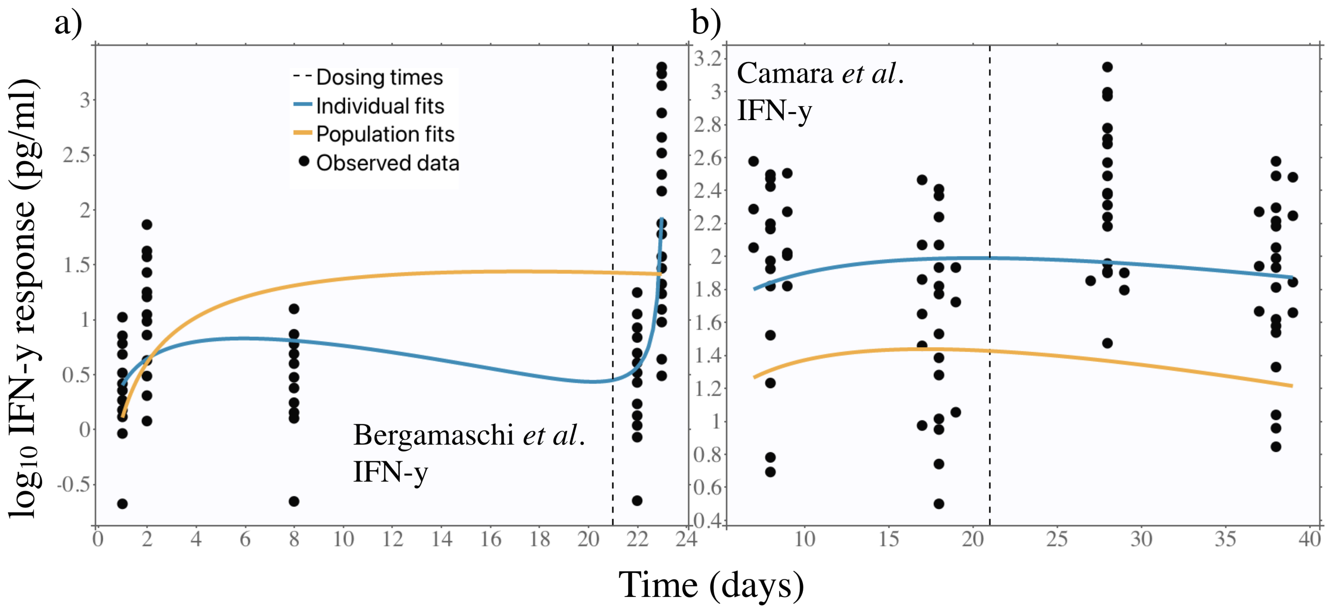

Figure S2: Individual fits to various IFN-γ data sets. References for the data set sources can be found in Table 1 of the main text, all individual fitted parameters for each fit can be found in Tables S3 and S4.

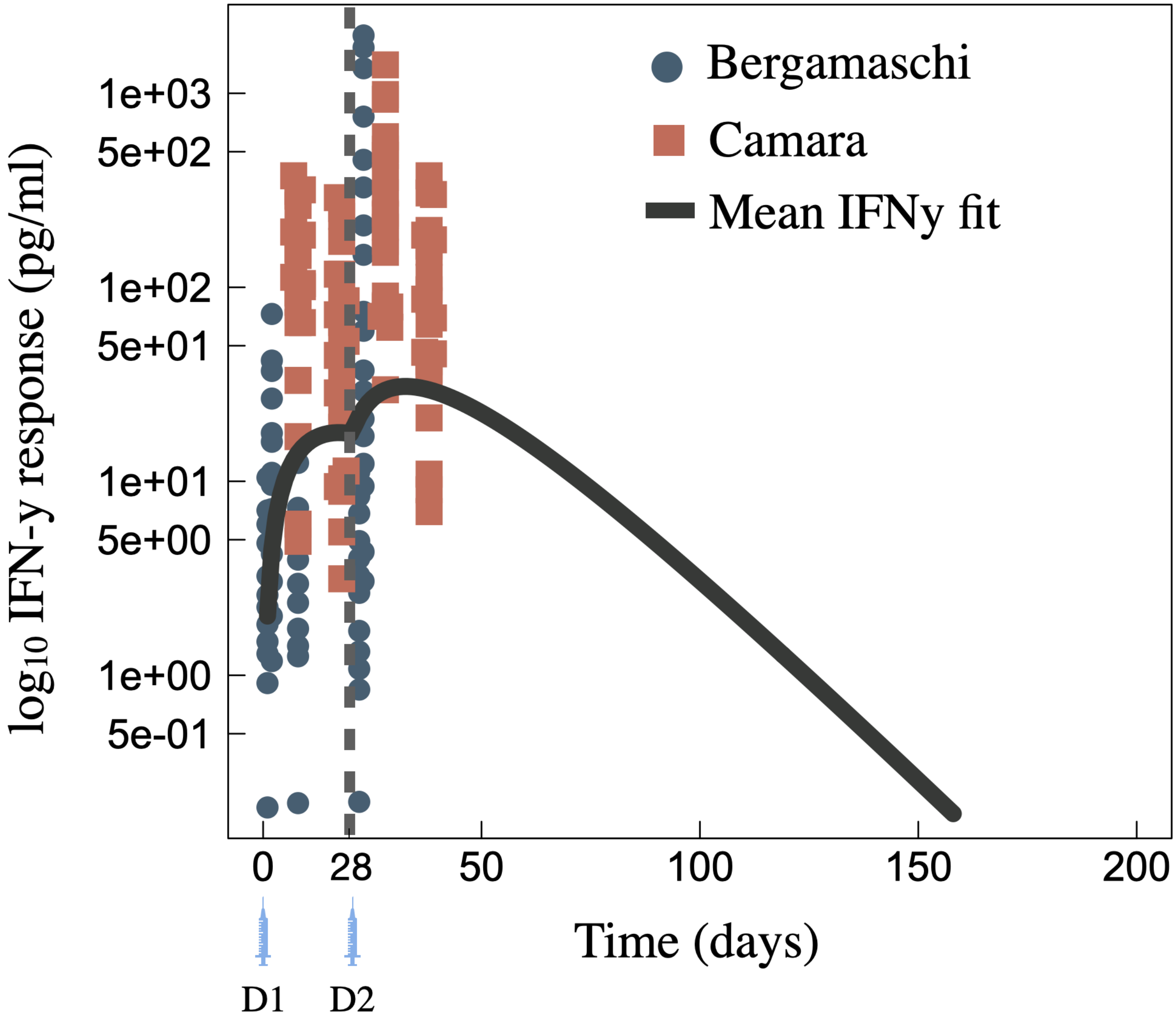

Figure S3: Mean IFNγ fit response to the Bergamaschi and Camara data sets.

3.4 Individual fits to Interleukin data sets

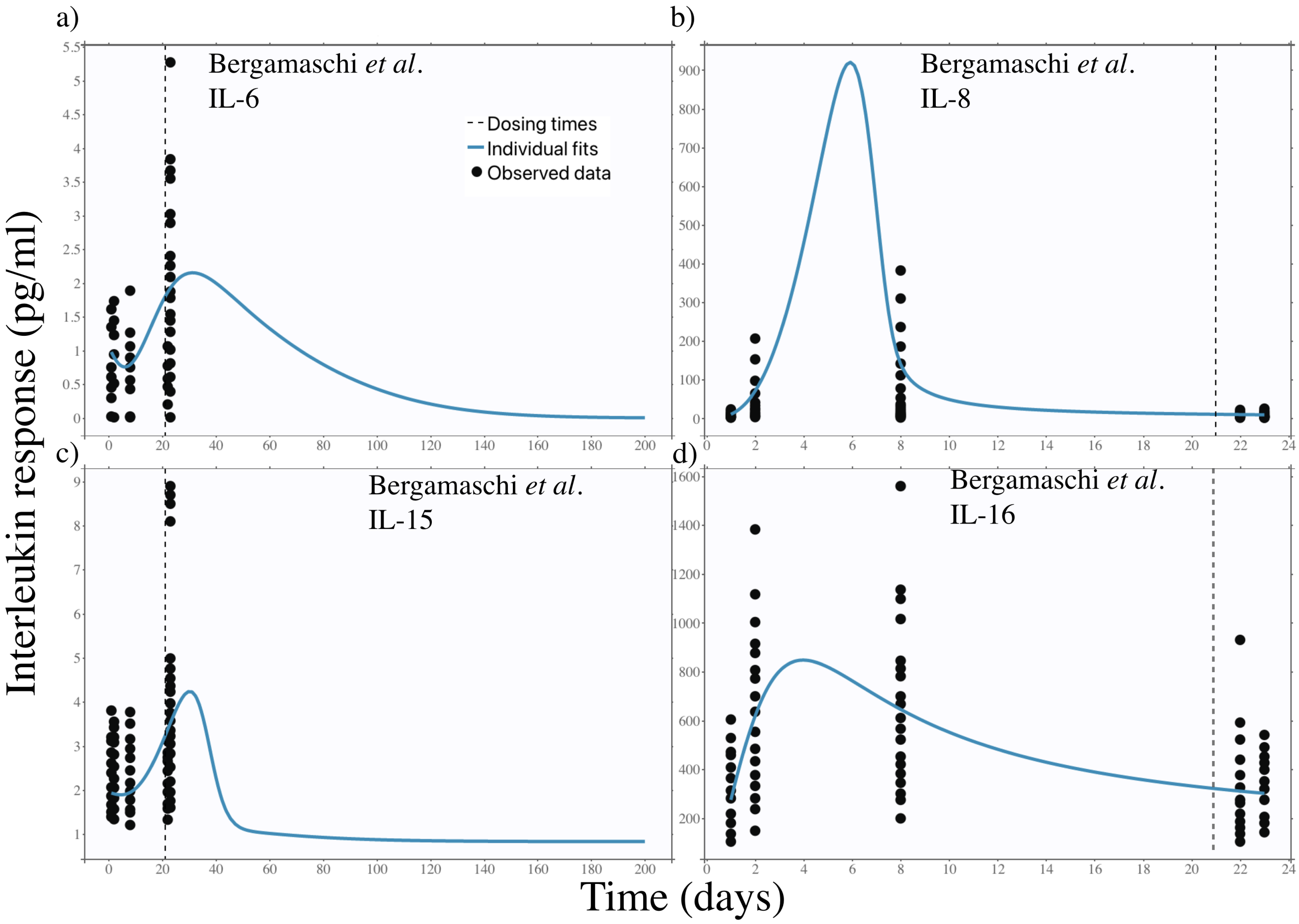

Figure S4: Individual fits to various interleukin data sets. References for the data set sources can be found in Table 1 of the main text, all individual fitted parameters for each fit can be found in Tables S3 and S4.

3.5 Goodness of fit predictive checks and parameter distributions

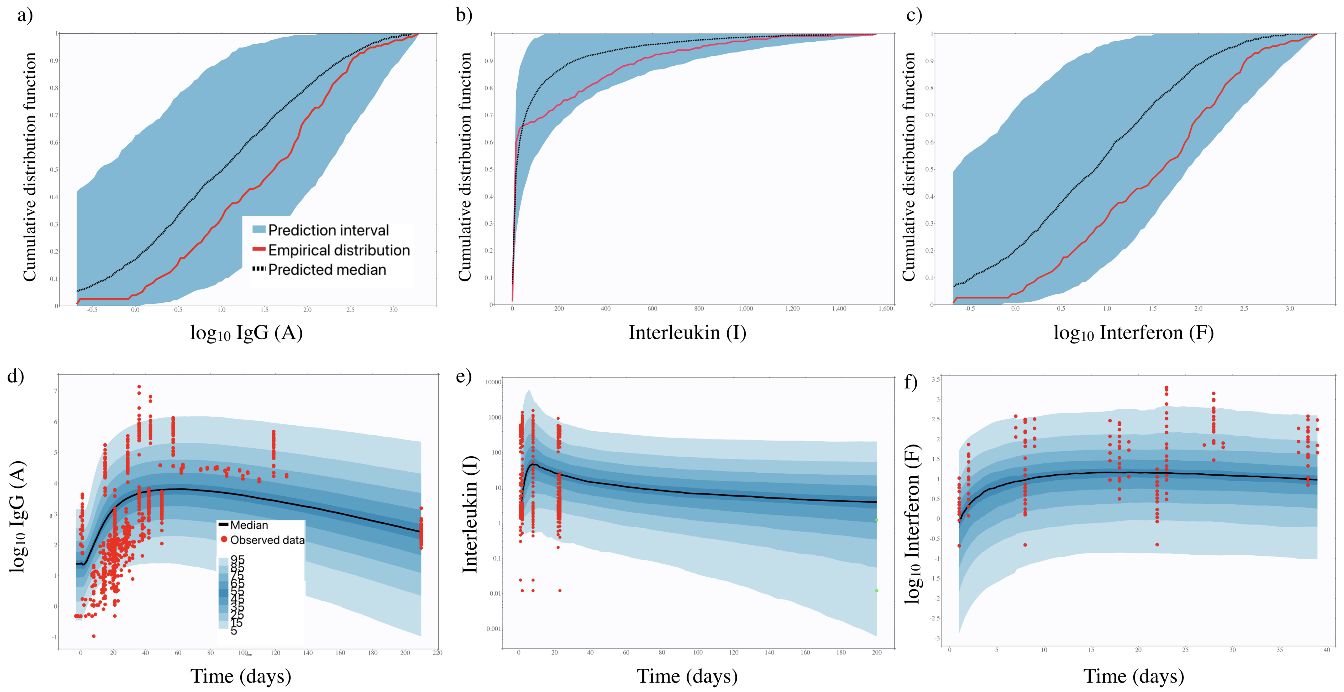

Figure S5: Predictive checks for two-dose mRNA results. a)-c) Numerical predictive checks for IgG, Interleukin, and Interferon, corresponding to fits to equations 9e, 9g, and 9h, respectively. d)-f) Prediction distributions for IgG, Interleukin, and Interferon as a function of time.

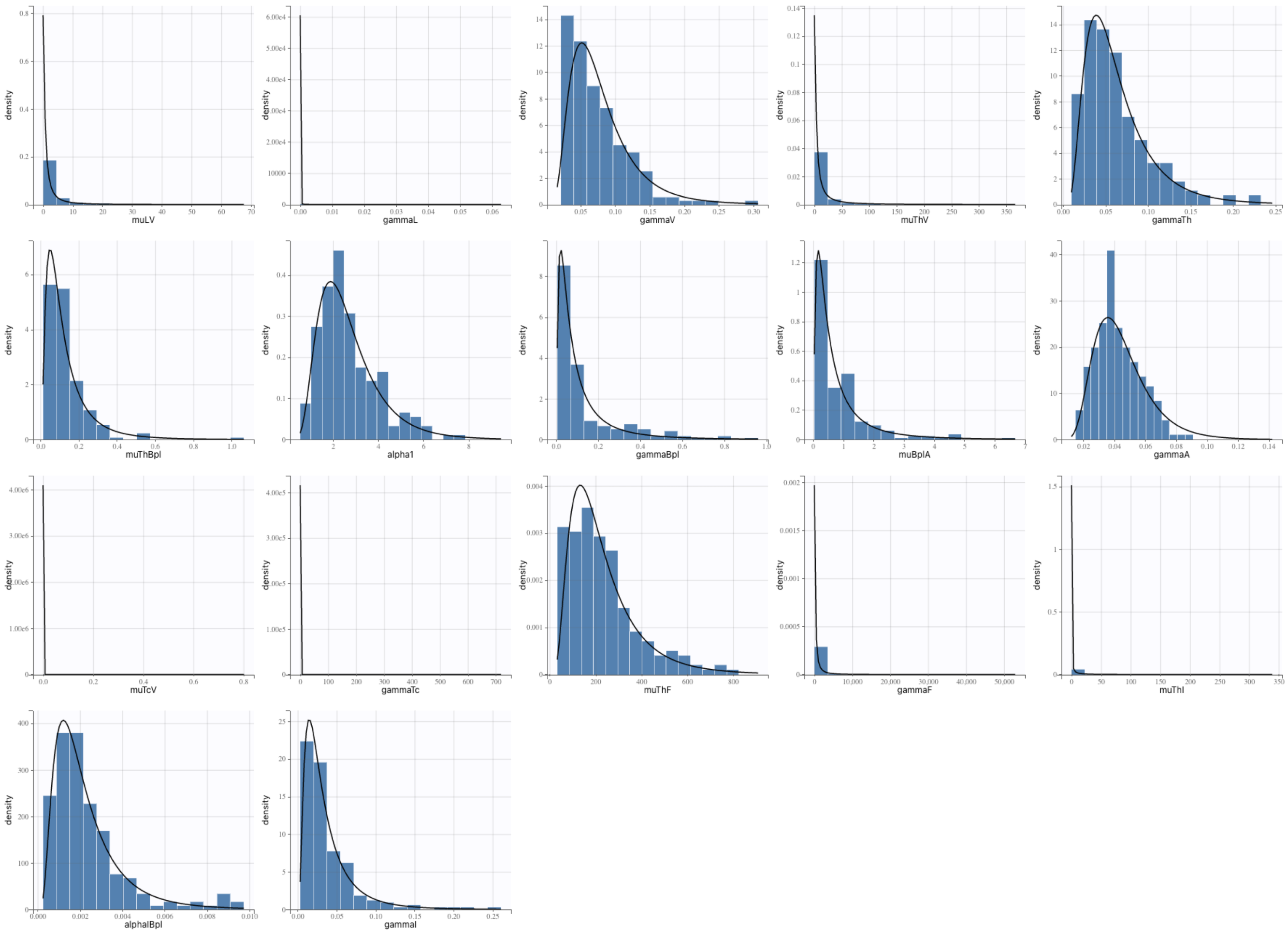

Figure S6: Monolix-determined fitted parameter distributions for all two standard dose fitted parameters.

3.6 Standardized random effects

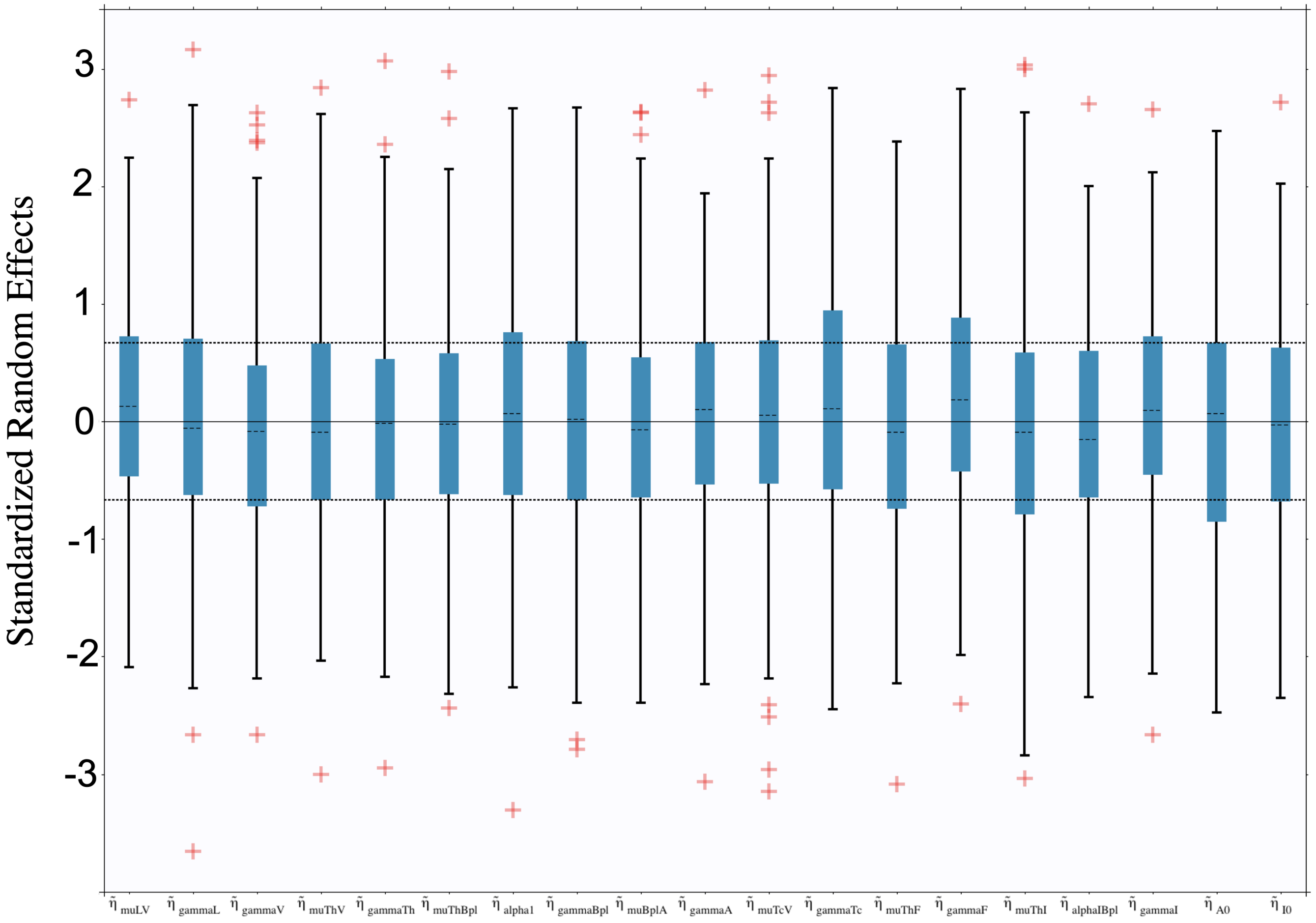

Figure S7: Boxplots of standardized random effects for all fitted model parameters for the two-dose mRNA data. The dashed line represents the median, the blue boxes represent the 25th and 75th percentiles, whiskers extend to extreme data points. Outliers are shown as red crosses.

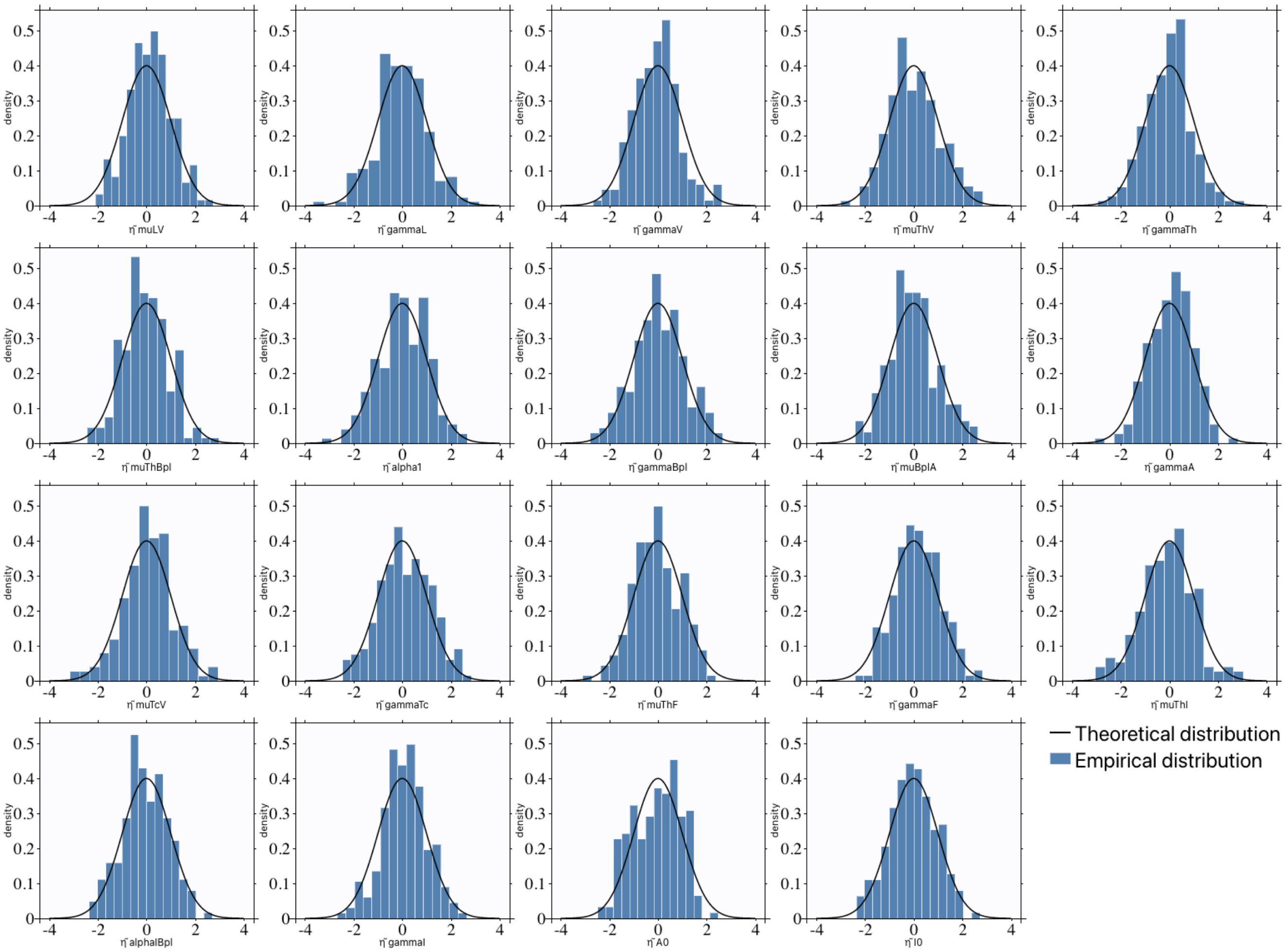

Figure S8: distributions of standardized random effects for all fitted model parameters for the two-dose mRNA data. The solid black line is an overlaid Guassian distribution.

4 Two low doses of mRNA-1273 vaccination

Two-low-dose mRNA-1273 data used in this work is sourced from Ref. [10]. This section contains tables summarizing the individual data set fitted parameters.

		Population fit values		
Parameter	Definition	Two standard doses	SD Random Effects	Comment
μ_{LV}	LNP absorption rate with antigen presenting cells	0.023	0.01	Fit
γ_L	LNP degradation rate	0.00026	2.52	Fit
γ_V	Antigen presenting cell death rate	0.098	0.056	Fit
μ_{TV}	CD4+ activation rate by vaccinated cells	3.86	0.027	Fit
γ_T	CD4+ natural death rate	0.055	N/A	Ref. [1]
μ_{TB}	Plasma B cell activation rate by CD4+ cells	2.48	0.02	Fit
α_{BI}	Plasma B cell stimulation by Interleukin	0.019	3.1	Fit
S_I	Plasma B cell duplication threshold due to Interleukin	1000	N/A	Fixed
γ_B	Plasma B cell natural death rate	0.072	0.034	Fit
μ_{BA}	Released antibody rate by plasma B cells	0.48	0.022	Fit
γ_A	Antibody natural degradation rate	0.067	0.05	Fit
μ_{CV}	CD8+ activation rate by vaccinated cells	0.00033	17.5	Fit
α_{CF}	CD8+ stimulation by IFN-γ	0.00096	N/A	Fit (no REs)
S_F	CD8+ duplication threshold due to IFN-γ	600	N/A	Fixed
γ_C	CD8+ natural death rate	0.01	9.29	Ref. [2]
μ_{TF}	IFN-γ stimulation rate by Thelper cells	0.22	27.7	Fit
α_{FC}	IFN-γ clearance by cytotoxic Tcells	0.000004	N/A	Fit (no REs)
γ_F	IFN-γ natural degradation rate	64.82	5.06	Fit
μ_{TI}	Interleukin secretion by CD4+ cells	0.000057	9.10	Fit
α_{IB}	Interleukin clearance by Plasma B cells	1.07	5.0	Fit
γ_I	Natural interleukin degradation rate	0.00001	36	Fit
A_0	Antibody initial condition	3.8	0.018	Fit
I_0	Interleukin initial condition	1.0	37.53	Fit
BIC	Bayesian Information Criteria	315		Fit
AIC	Akaike Information Criteria	252		Fit

Table S5: Model parameters definition and population fitted values for two low doses of mRNA-1273. The dosing times are separated by 28 days for mRNA-1273.

4.1 Model parameter population fits and individual data set fitted values

Data set ID	Figure from paper	μ_{LV}	γ_L	γ_V	μ_{TV}	γ_T	μ_{TB}	α_{BI}	s_I	γ_B	μ_{BA}	γ_A
Mateus *et al.* [10]	Fig. 1a (Spike IgG)	0.023	0.00027	0.099	3.85	0.056	2.48	0.019	1003.37	0.073	0.48	0.068
Mateus *et al.* [10]	Fig. 1b (RBD IgG)	0.023	0.00025	0.097	3.87	0.054	2.49	0.019	1002.3	0.071	0.48	0.066

Table S6: Individual fit values for to the two low doses of mRNA-1273. This table contains all fitted parameters from equations 9a, 9b, 9c, 9d, and 9e.

Data set ID	μ_{CV}	α_{CF}	S_F	γ_C	μ_{TF}	α_{FC}	γ_F	μ_{TI}	α_{IB}	γ_I
Mateus *et al.* [10]	0.00033	0.00096	608.69	0.0099	0.21	0.000004	64.94	0.000057	1.07	0.0000074
Mateus *et al.* [10]	0.00033	0.00096	604.89	0.0099	0.2	0.000004	64.94	0.000056	1.07	0.0000074

Table S7: Individual fit values for to the two low doses of mRNA-1273. This table contains all fitted parameters from equations 9f, 9g and 9h.

4.2 Individual fits to IgG data sets

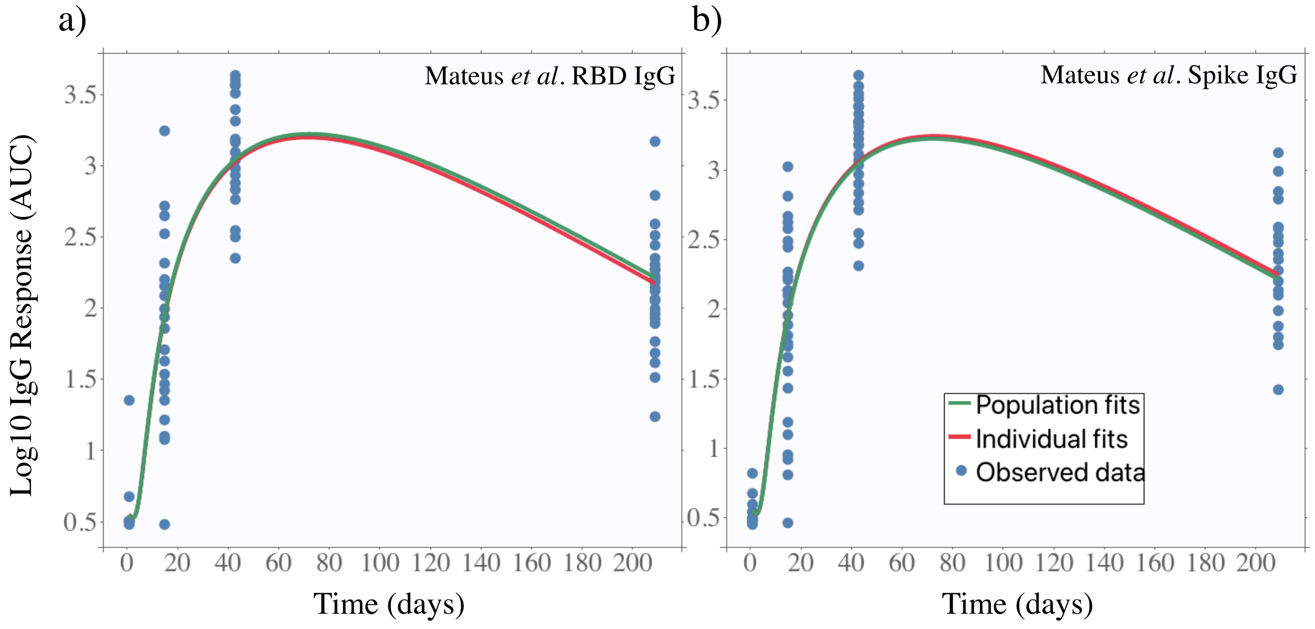

Figure S9: Individual fits to the two low doses of mRNA-1273. All individual fitted parameter values can be found in Tables S6 and S7.

4.3 Goodness of fit predictive checks

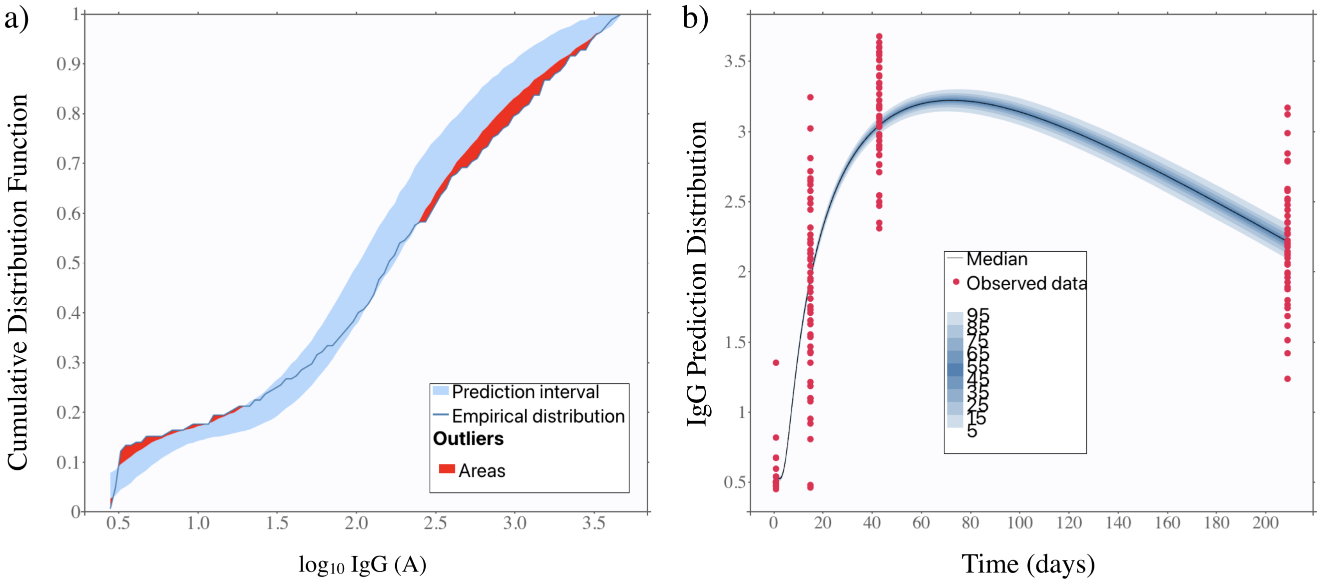

Figure S10: Predictive checks for fits to two low doses of mRNA-1273. a) Numerical predictive checks for the IgG data sets. b) Prediction distributions for the IgG fits as a function of time.

4.4 Standardized random effects

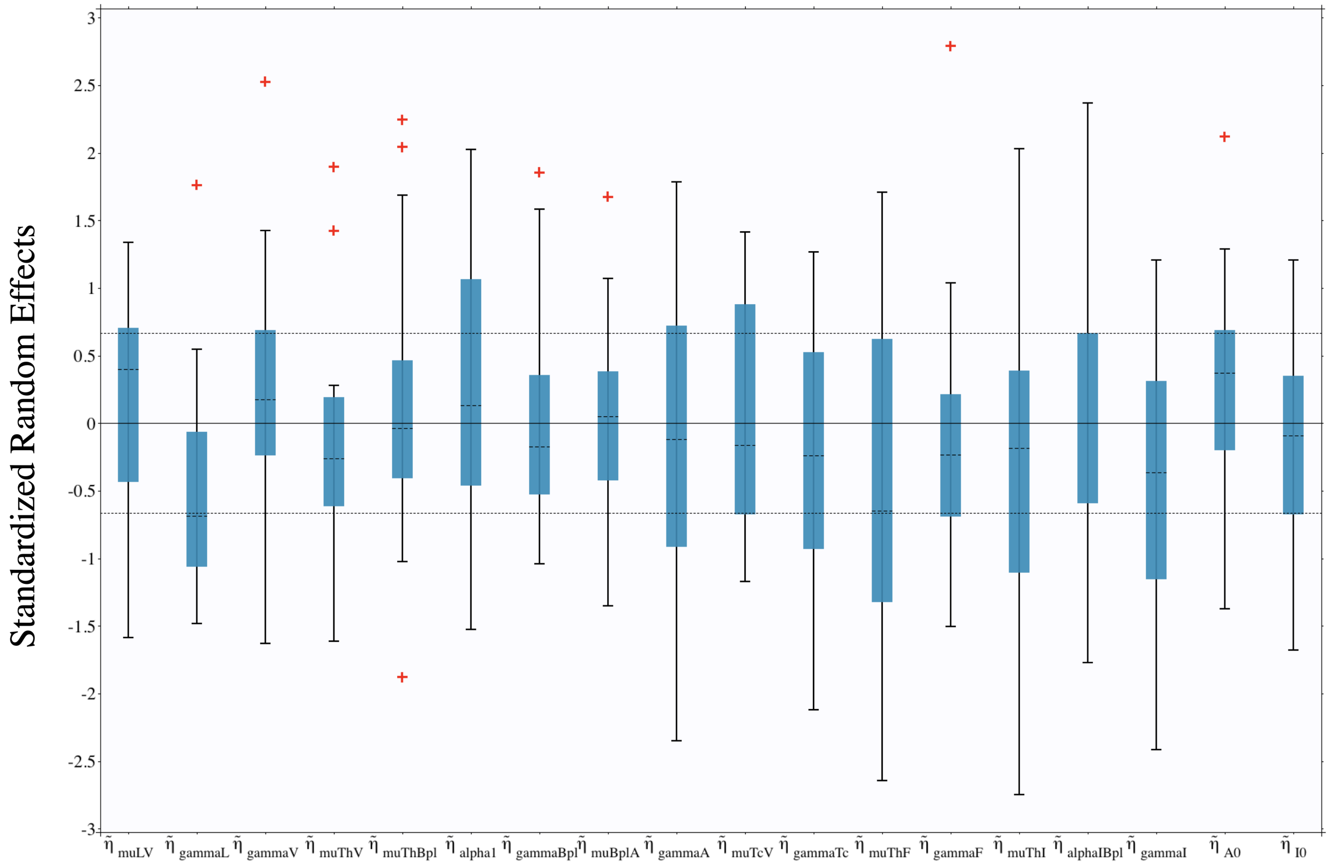

Figure S11: Boxplots of standardized random effects for all fitted model parameters for the low dose mRNA data. The dashed line represents the median, the blue boxes represent the 25th and 75th percentiles, whiskers extend to extreme data points. Outliers are shown as red crosses.

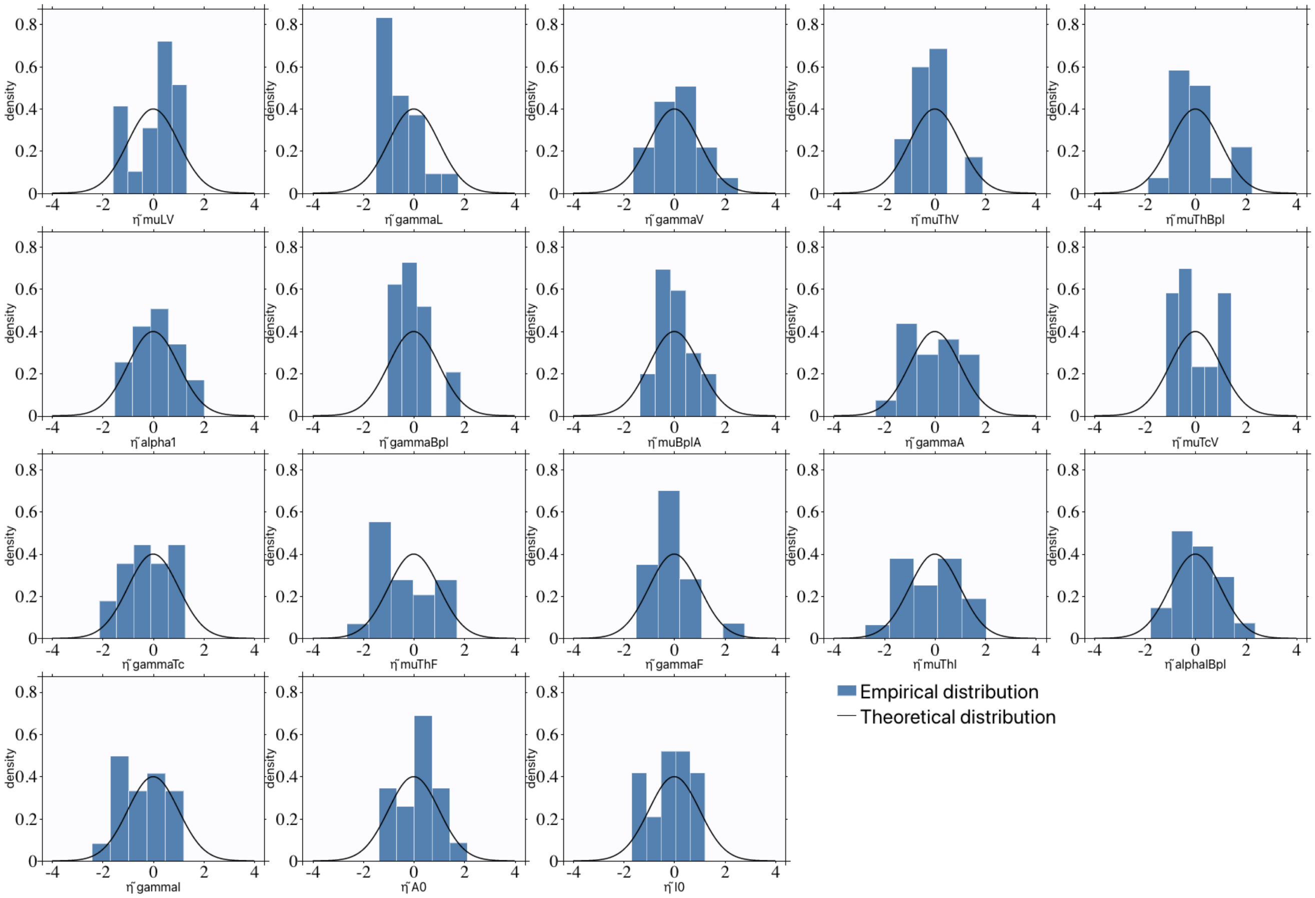

Figure S12: distributions of standardized random effects for all fitted model parameters for the low dose mRNA data. The solid black line is an overlaid Guassian distribution.

5 Sensitivity Analysis

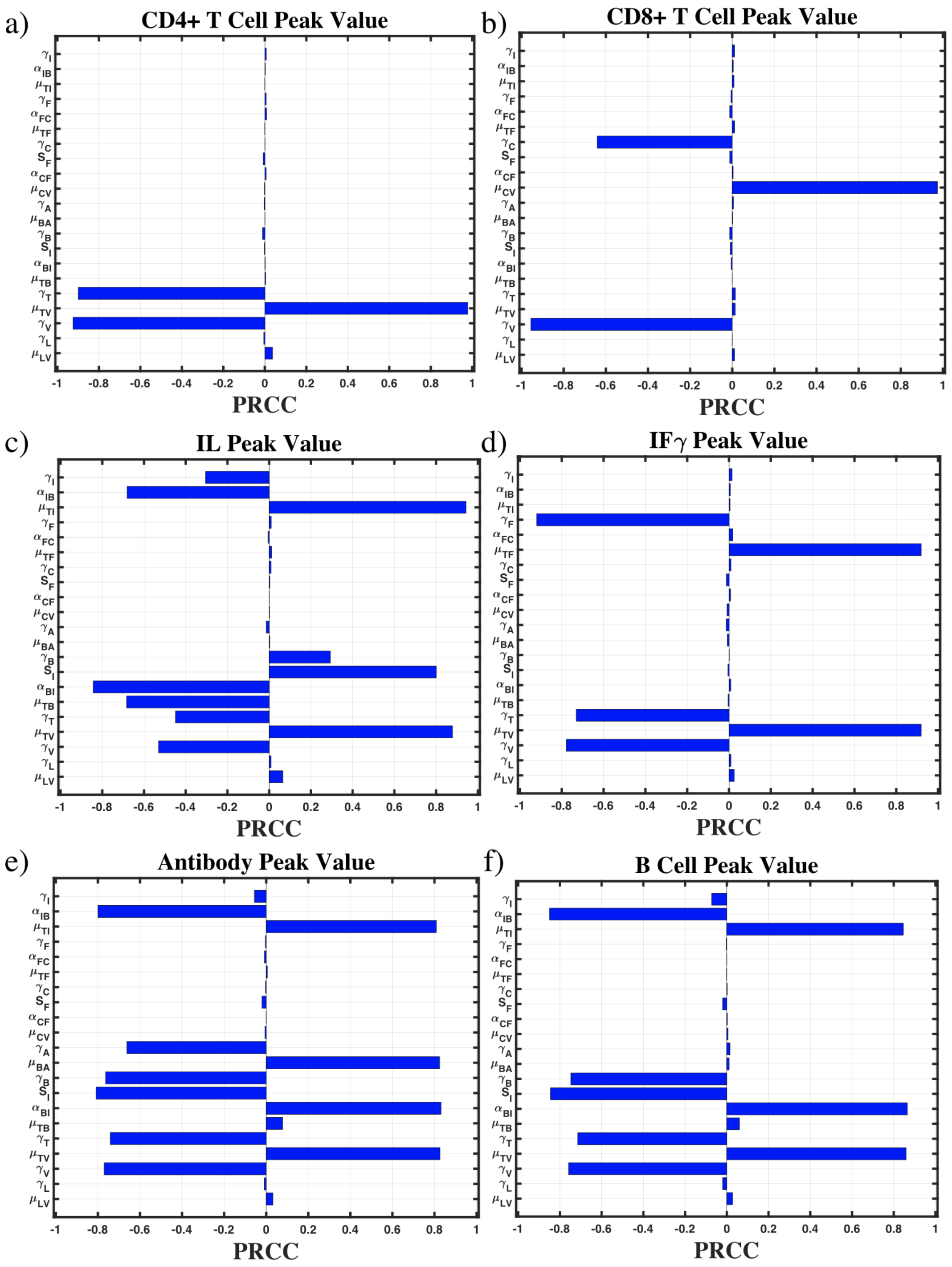

Figure S13: PRCC sensitivity analysis. PRCC analysis is performed on model parameters where the peak value of each state variable is selected as the model output. Panels a-f show the sensitivity analysis for CD4+ T cells (T), CD8+ T cells (C), Interleukin (I), IFNγ (F), Antibody (A), and Plasma B cells (B), respectively.

6 Model complexity reduction

The full model (Eq.9) was derived following a biologically consistent approach to the immune response upon receiving an LNP mRNA based vaccine. On the surface it appears to be a complex 8 equation coupled model and thus parameter estimation would lead to coupled overfitting complications. However, the model is actually only weakly coupled through an effective activation-inhibition mechanism between plasma B-cells and interleukin.

We first note the immediate decoupling in the model. The first equations, Eq.9a-c, for L, V, and T are a cascade of linear equations which can be solved in sequence (and analytically). Furthermore, the equation for A depends on, but is decoupled from, B and thus can be solved once the solution for B is independently known,

$$A = \mu_{BA} \mathrm{e}^{-\gamma_A t} \int_0^t B(s) \mathrm{e}^{\gamma_A s} \mathrm{d}s. \tag{1}$$

Thus, there are two sets of equations remaining, a coupled system for B and I and a coupled system for C and F. The parameter fitting in Table S4 demonstrates that the CD8$^+$ cell-mediated removal of IFN-γ is eclipsed by its natural decay rate. This suggests that the term associated to α_{FC} can be ignored. Furthermore, the production of IFN-γ, μ_{TF} is comparable to the decay rate γ_F which are both very quick. This suggests a quasi-steady balance of production and decay and that

$$F = \frac{\mu_{TF}}{\gamma_F} T. \tag{2}$$

If the CD8$^+$ cell-mediated removal of IFN-γ is small then it is reasonable to suggest that the IFN-γ mediated production of CD8$^+$ cells is also negligible which is supported by the small value of α_{CF} compared to γ_C and μ_{CV} in Table S4. This means that the equation for C also decouples and follows a similar solution to that of the CD4$^+$ T-cells,

$$\frac{\mathrm{d}C}{\mathrm{d}t} = \mu_{CV} V - \gamma_C C. \tag{3}$$

Therefore, with these assumptions our eight equation model really becomes a two equation non-linear coupled system for plasma B-cells and interleukin. However, if we anticipate that the response of the vaccine is mostly transient then we are likely not near the threshold required for saturation effects. This suggests that the parameter s_I is not needed for model consistency. This parameter was arbitrarily chosen and its value being unimportant demonstrate strong model robustness. Overall, we then expect the reduced eight equation model is equivalent to solving

$$\frac{\mathrm{d}B}{\mathrm{d}t} = \mu_{TB} T + \alpha_{BI} I B - \gamma_B B \tag{4a}$$

$$\frac{\mathrm{d}I}{\mathrm{d}t} = \mu_{TI} T - \alpha_{IB} I B - \gamma_I I. \tag{4b}$$

Fig.S14 shows the full solution (solid blue) to the full model (Eq.9) and the reductions (dashed red) Eq.1 for A, Eq.2 for F, and Eq.3 for C as well as the analytically obtained decoupled solutions for L, V, and T. We also

demonstrate in Figure Fig.S14h that indeed the saturation parameter being removed does not change the Interleukin response with the red dashed curve having the parameter excluded from the model.

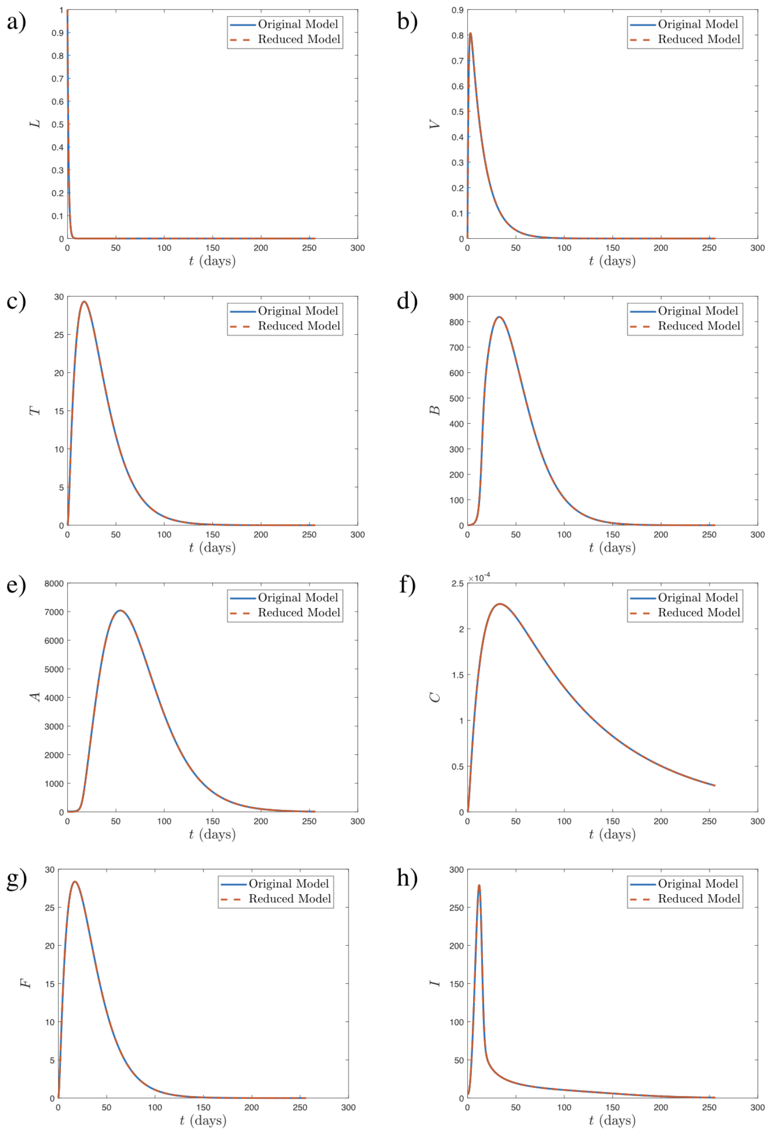

Figure S14: Comparison of the full model (Eq.9) (in solid blue) and the various reduction assumptions (1), (3), (2), and (4) as well as the analytic reductions for L, V, and T (in dashed red). The parameter sets chosen were the population fits in Table S2.

References

[1] P. Cao, Z. Wang, A. W. C. Yan, J. McVernon, J. Xu, J. M. Heffernan, K. Kedzierska, and J. M. McCaw, "On the Role of CD8+ T Cells in Determining Recovery Time from Influenza Virus Infection," *Frontiers in Immunology*, vol. 7, p. 611, 2016.

[2] Z. Wang, Y. Wan, C. Qiu, S. Quiñones-Parra, Z. Zhu, L. Loh, D. Tian, Y. Ren, Y. Hu, X. Zhang, P. G. Thomas, M. Inouye, P. C. Doherty, K. Kedzierska, and J. Xu, "Recovery from severe H7N9 disease is associated with diverse response mechanisms dominated by CD8+ T cells," *Nature Communications*, vol. 6, no. 1, p. 6833, 2015.

[3] R. R. Goel, S. A. Apostolidis, M. M. Painter, D. Mathew, A. Pattekar, O. Kuthuru, S. Gouma, P. Hicks, W. Meng, A. M. Rosenfeld, S. Dysinger, K. A. Lundgreen, L. Kuri-Cervantes, S. Adamski, A. Hicks, S. Korte, D. A. Oldridge, A. E. Baxter, J. R. Giles, M. E. Weirick, C. M. McAllister, J. Dougherty, S. Long, K. D'Andrea, J. T. Hamilton, M. R. Betts, E. T. Luning Prak, P. Bates, S. E. Hensley, A. R. Greenplate, and E. J. Wherry, "Distinct antibody and memory B cell responses in SARS-CoV-2 naïve and recovered individuals following mRNA vaccination.," *Science immunology*, vol. 6, no. 58, pp. 1–20, 2021.

[4] M. V. Stankov, A. Cossmann, A. Bonifacius, A. Dopfer-Jablonka, G. M. Ramos, N. Gödecke, A. Z. Scharff, C. Happle, A.-L. Boeck, A. T. Tran, I. Pink, M. M. Hoeper, R. Blasczyk, M. S. Winkler, I. Nehlmeier, A. Kempf, H. Hofmann-Winkler, M. Hoffmann, B. Eiz-Vesper, S. Pöhlmann, and G. M. N. Behrens, "Humoral and Cellular Immune Responses Against Severe Acute Respiratory Syndrome Coronavirus 2 Variants and Human Coronaviruses After Single BNT162b2 Vaccination," *Clinical Infectious Diseases*, pp. 1–9, 2021.

[5] C. Bergamaschi, E. Terpos, M. Rosati, M. Angel, J. Bear, D. Stellas, S. Karaliota, F. Apostolakou, T. Bagratuni, D. Patseas, S. Gumeni, I. P. Trougakos, M. A. Dimopoulos, B. K. Felber, and G. N. Pavlakis, "Systemic IL-15, IFN-γ, and IP-10/CXCL10 signature associated with effective immune response to SARS-CoV-2 in BNT162b2 mRNA vaccine recipients," *Cell Reports*, vol. 36, no. 6, p. 109504, 2021.

[6] C. Camara, D. Lozano-Ojalvo, E. Lopez-Granados, E. Paz-Artal, M. Pion, R. Correa-Rocha, A. Ortiz, M. Lopez-Hoyos, M. Erro Iribarren, J. Portoles, P. Portoles, M. Perez-Olmeda, J. Oteo, C. Berin, E. Guccione, A. Bertoletti, and J. Ochando, "Differential effects of the second SARS-CoV-2 mRNA vaccine dose on T cell immunity in naïve and COVID-19 recovered individuals," *bioRxiv*, p. 2021.03.22.436441, 2021.

[7] A. T. Widge and N. Rouphael, "Durability of Responses after SARS-CoV-2 mRNA-1273 Vaccination," *The New England journal of medicine*, vol. 384, no. 1, pp. 7–10, 2021.

[8] Z. Wang, F. Schmidt, Y. Weisblum, F. Muecksch, C. O. Barnes, S. Finkin, D. Schaefer-Babajew, M. Cipolla, C. Gaebler, J. A. Lieberman, T. Y. Oliveira, Z. Yang, M. E. Abernathy, K. E. Huey-Tubman, A. Hurley, M. Turroja, K. A. West, K. Gordon, K. G. Millard, V. Ramos, J. D. Silva, J. Xu, R. A. Colbert, R. Patel, J. Dizon, C. Unson-O'Brien, I. Shimeliovich, A. Gazumyan, M. Caskey, P. J. Bjorkman, R. Casellas, T. Hatziioannou, P. D. Bieniasz, and M. C. Nussenzweig, "mRNA vaccine-elicited antibodies to SARS-CoV-2 and circulating variants," *Nature*, vol. 592, no. April, 2021.

[9] M. S. Suthar, P. S. Arunachalam, M. Hu, N. Reis, M. Trisal, O. Raeber, S. Chinthrajah, M. E. Davis-Gardner, K. Manning, P. Mudvari, E. Boritz, S. Godbole, A. R. Henry, D. C. Douek, P. Halfmann, Y. Kawaoka, S. D. Boyd, M. M. Davis, V. I. Zarnitsyna, K. Nadeau, and B. Pulendran, "Durability of immune responses to the BNT162b2 mRNA vaccine," *Med*, vol. 3, no. 1, pp. 25–27, 2022.

[10] J. Mateus, J. M. Dan, Z. Zhang, C. R. Moderbacher, M. Lammers, B. Goodwin, A. Sette, S. Crotty, and D. Weiskopf, "Low-dose mRNA-1273 COVID-19 vaccine generates durable memory enhanced by cross-reactive T cells," *Science*, vol. 374, no. 6566, p. eabj9853, 2021.
